# Occurrence and Distribution of Environmental *Pseudomonas aeruginosa* From Hospitals in Bangladesh Reveals Diverse Strain Families, Multidrug Resistance, and Biofilm Formation

**DOI:** 10.1111/1758-2229.70391

**Published:** 2026-07-24

**Authors:** S. M. Kador, Tanay Chakrovarty, Khandaker Adil, Md. Mustak Ahmed, Md. Mohsin Kobir, Md. Hasanuzzaman, Mousufa Akter, Tanzima Sharmim Taowhid, Md. Tanvir Islam, Ovinu Kibria Islam

**Affiliations:** ^1^ Department of Microbiology Jashore University of Science and Technology Jashore Bangladesh

**Keywords:** antimicrobial resistance genes, Bangladesh, biofilm, hospital environment, integrons, multidrug resistance, *Pseudomonas aeruginosa*

## Abstract

*Pseudomonas aeruginosa* is a major nosocomial pathogen capable of developing multidrug resistance, forming biofilms, and disseminating antimicrobial resistance genes via mobile genetic elements. This study investigated the occurrence, antimicrobial resistance, *lecB* sequence diversity, and biofilm‐forming capacity of environmental *P. aeruginosa* isolates from three hospitals in Jashore, Bangladesh. Among 231 environmental samples collected from 29 sites in high‐risk hospital units, 36 isolates were confirmed by *lecB* gene sequencing. Sequence analysis classified 77.8% of isolates as PAO1‐like and 22.2% as PA14‐like, indicating genetic diversity. Biofilm formation was detected in 97.2% of isolates, with no significant difference between strain families. Phenotypic susceptibility testing revealed widespread multidrug resistance, with the highest resistance to ceftazidime (97.2%) and tetracycline (91.7%). Two operation theatre isolates (A40 and 2SW) exhibited extensive drug resistance within the tested antimicrobial panel. Polymerase chain reaction detected *blaSHV* (55.6%), *floR* (33.3%), *aac*(*6′*)*‐Ib* (33.3%), *blaTEM* (27.8%), and *blaOXA‐48* (11.1%), while class 1 integrons occurred in 55.6% of isolates and were associated with *floR* and *blaTEM*. Whole‐genome sequencing identified intrinsic resistance determinants and the mobile resistance genes *tmexCD‐toprJ* and *crpP*. These findings highlight genetically diverse, multidrug‐resistant, integron‐positive, biofilm‐forming *P. aeruginosa* in Bangladeshi hospital environments, underscoring the need for routine environmental surveillance and strengthened infection prevention.

## Graphical abstract

Environmental reservoirs in Bangladeshi hospitals harbor phylogenetically diverse, multidrug‐resistant *Pseudomonas aeruginosa* with extensive biofilm‐forming capacity and class 1 integron‐associated resistance genes. These findings highlight hospital environments as persistent sources of antimicrobial resistance dissemination and underscore the need for routine environmental surveillance and strengthened infection control.

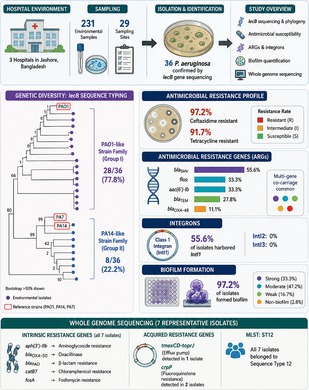

## 1 Introduction

*Pseudomonas aeruginosa* is a Gram‐negative, non‐fermenting opportunistic pathogen consistently ranked among the most problematic nosocomial bacteria worldwide by the World Health Organization (WHO) and the Centers for Disease Control and Prevention (CDC) (Shrivastava et al. 2018; Kadri 2020). Its clinical prominence stems from intrinsic physiological resilience, metabolic versatility, and an extraordinary capacity to acquire resistance determinants against virtually all clinically available antibiotic classes (Botelho et al. 2019). It is the leading causative agent of ventilator‐associated pneumonia, catheter‐associated urinary tract infections, surgical site infections, and burn wound infections, responsible for disproportionately high morbidity and mortality in immunocompromised and critically ill patients (Moradali et al. 2017; Nathwani et al. 2014). The global burden of multidrug‐resistant (MDR) *P. aeruginosa* is substantial, with hospital‐acquired infections associated with crude mortality rates of 30%–60% in high‐acuity settings and increasingly restricted therapeutic options as carbapenem resistance escalates (Kollef et al. 2012; Lister et al. 2009). Beyond clinical isolates, hospital environmental surfaces, water systems, and equipment represent underappreciated reservoirs where resistance determinants accumulate and are amplified before transmission to patients.

The antimicrobial resistance architecture of *P. aeruginosa* is both multilayered and synergistic. Intrinsic mechanisms including low outer membrane permeability, constitutive MexAB‐OprM efflux pump expression, and inducible *AmpC* β‐lactamase activity are compounded by acquired resistance through chromosomal mutations and horizontal gene transfer via mobile genetic elements (MGEs) (Poole 2011; Mesaros et al. 2007; Cabot et al. 2012). Among these, integron‐mediated gene transfer is particularly consequential: class 1 integrons capture and disseminate arrays of resistance gene cassettes conferring simultaneous resistance to multiple antibiotic classes, and their carriage is consistently associated with MDR phenotypes across diverse hospital settings (Stokes and Hall 1989; Khademi et al. 2021) Additionally, *P. aeruginosa* forms robust biofilms on biotic and abiotic surfaces, enabling it to tolerate antibiotic concentrations up to 1000‐fold higher than planktonic cells while simultaneously facilitating horizontal gene transfer between co‐resident strains (Sharma et al. 2019; Ciofu et al. 2022; Zafer et al. 2024).

The diversity and pathogenicity of *Pseudomonas aeruginosa* are commonly investigated using the reference strains PAO1 and PA14. Although these strains belong to the same species, they differ in virulence characteristics and in the amino acid sequence of the fucose‐binding lectin LecB, a key adhesin involved in host interaction, bacterial adhesion, and biofilm formation. Sommer et al. demonstrated that sequence variation in LecB influences carbohydrate‐binding affinity while maintaining similar glycan‐binding specificity, suggesting functional differences between the PAO1 and PA14 LecB variants. Furthermore, they showed that *lecB* sequence variation closely correlates with broader genomic differences and can serve as a genetic marker for classifying *P. aeruginosa* isolates into the two major strain families, PAO1‐like and PA14‐like (Sommer et al. 2016; Islam et al. 2023).

The hospital environment encompassing surfaces, water systems, medical equipment, and high‐traffic areas serves as an active reservoir for MDR *P. aeruginosa* (Trautmann et al. 2005; de Abreu et al. 2014). Environmental strains recovered from hospital sinks, drains, and floors have been shown to be phylogenetically indistinguishable from clinical isolates causing infections in the same facilities, directly implicating environmental reservoirs in nosocomial transmission (Römling et al. 1994). Yet comprehensive environmental characterization integrating resistance profiling, ARG and MGE screening, whole genome sequencing, and biofilm quantification remains underexplored, particularly in resource‐limited South Asian healthcare settings where stewardship infrastructure is constrained (Pittet et al. 2008; Laxminarayan et al. 2013). Bangladesh exemplifies this gap: *P. aeruginosa* resistance rates in Bangladeshi hospitals rank among the highest in South Asia, with ceftazidime resistance exceeding 76% and carbapenem resistance increasingly driven by metallo‐β‐lactamase production, against a backdrop of unregulated antibiotic prescribing and inadequate infection control (Ramatla et al. 2025; Saha et al. 2022; Sulis et al. 2020).

The diversity and pathogenicity of *P. aeruginosa* are frequently studied using two reference strains, PAO1 and PA14. Despite their close relationship, they differ in pathogenicity and the sequence of the fucose‐binding lectin LecB, a crucial adhesin involved in host interaction and biofilm formation. These differences affect ligand binding and biofilm stability across lineages. Additionally, variation in *lecB* sequences was suggested as a strain‐family classification marker (Sommer et al. 2016; Islam et al. 2023). Unlike previous Bangladeshi studies that have focused primarily on clinical *P. aeruginosa* isolates (Ramatla et al. 2025; Saha et al. 2022; Rashid et al. 2007), the present study characterizes the environmental resistome, integron profile, and biofilm phenotype of *P. aeruginosa* across multiple hospital microenvironments simultaneously, providing the first integrated molecular‐epidemiological picture of environmental *P. aeruginosa* in this setting.

## 2 Methodology

### 2.1 Study Site, Sample Collection, and Bacterial Isolation

**FIGURE 1:**
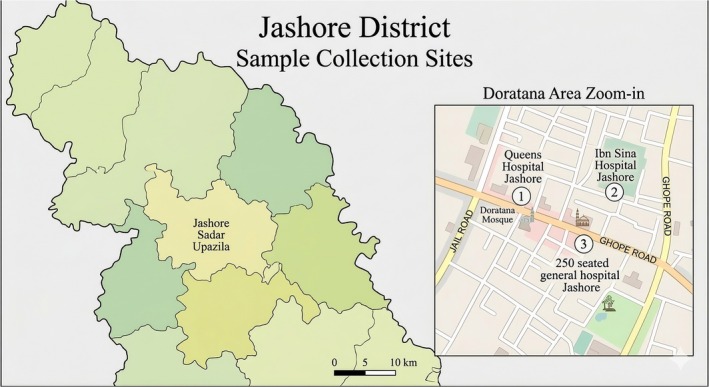
Map of sample collection sites in Jashore District, Bangladesh. The left panel shows the administrative map of Jashore District, with Jashore Sadar Upazila highlighted. The right panel shows a street‐level zoom‐in of the Doratana area, indicating the three hospital sampling sites: (1) Queens Hospital Jashore, (2) Ibn Sina Hospital Jashore, and (3) 250‐Seated General Hospital Jashore, situated along Jail Road and Ghope Road in the Doratana locality. Scale bar = 10 km.

This study was conducted between April 2025 and November 2025 at three hospitals located within the Doratana area of Jashore Sadar Upazila, Jashore district, Bangladesh: Queens Hospital Jashore, Ibn Sina Hospital Jashore, and the 250‐Seated General Hospital Jashore (Figure 1). Environmental surface and water samples were collected from hospital premises across two successive sampling rounds: the first during April–August 2025 and the second during August–November 2025. Detailed information on sampling periods and site‐wise sample distribution is provided in Table S1. Samples were collected in accordance with standard microbiological sampling procedures, with the knowledge and cooperation of the respective hospital authorities. A total of 231 samples were collected aseptically from 29 distinct sampling sites spanning high‐risk clinical units and general hospital areas, including the Operation Theatre, Burn Unit, ICU, Surgical Unit, Paediatric Ward, Wound Patients Ward, Male and Female Wards, Toilets, Drain, Ambulance, and Corridor, among others (Table 1). Sampling sites were selected using a risk‐based convenience sampling approach, prioritizing high‐risk clinical units known to harbor nosocomial pathogens alongside general‐access areas to capture the full within‐hospital environmental spectrum. Surface swabs were collected using sterile cotton swabs moistened with normal saline, while water and liquid samples were collected directly into sterile containers. All samples were stored at 4°C immediately after collection and transported to the laboratory within 1–2 h for processing.

**TABLE 1:** Sample collection sites, number of samples collected, and confirmed *P. aeruginosa* isolates per site across three hospitals in Jashore District, Bangladesh.

Sl No.	Sampling site	Sample count	*P. aeruginosa* isolates	Isolate ID*
1	Surgical unit	21	1	*PD*
2	I.C.U unit	5	0	
3	Pneumonia ward	9	2	7PWW, PWW
4	Pathology unit	15	1	***9PUF***
5	Kitchen water	6	0	
6	Corridor	7	0	
7	Outdoor unit	5	2	***2OB***, OB
8	Autoclave room	2	0	
9	Male ward	22	1	15MD
10	Ambulance	2	2	15AB, A1
11	Toilet	11	2	MTW, ***FT***
12	Toilet Water	7	2	3TDW, TW
13	Female ward	5	2	***FWD***, FW
14	Gynae ward	6	1	3GWF
15	Wound patients ward	6	3	1WPD, WPF, ***WPF1***
16	Drain	5	2	DW, HD
17	Kidney patients ward	3	1	2KW
18	Burn unit	4	4	13BWF, BW1, BW2, ***BW3***
19	Diabetes ward	3	0	
20	Asthma ward	2	1	A26
21	Plaster room	4	0	
22	Dustbin	5	0	
23	Entrance	19	0	
24	X‐Ray	4	0	
25	Operation Theatre	15	6	A40, 2OTI, 2SW, 3OTF, OTW, SW
26	Lift	7	0	
27	Stretcher	3	0	
28	Paediatric Ward	17	2	17CW1, CWF2
29	CCU	11	1	CCUW
	***Total***	**231**	**36**	

*Note:* A total of 231 environmental samples were collected from 29 distinct sampling sites spanning high‐risk clinical units and general hospital areas. The table lists each sampling site, the total number of samples collected, the number of confirmed *P. aeruginosa* isolates recovered, and their corresponding isolate identifiers. Isolate IDs shown in bold italics were selected for whole genome sequencing (WGS) based on diversity of sampling origin and resistance profile.

* Isolate IDs shown in bold and italics were subjected to whole genome sequencing.

Samples were inoculated onto Cetrimide Agar, a selective medium for *P. aeruginosa* (Lowbury and Collins 1955), and incubated at 37°C for 24–48 h. Presumptive colonies exhibiting characteristic morphology, pigmentation, and fruity odour were subjected to Gram staining and a panel of biochemical tests including oxidase, catalase, indole production, citrate utilization, and sugar fermentation tests for presumptive identification of the isolates (Figures S1–S3) (Murray et al. 1995). A total of 114 bacterial isolates were provisionally identified as *P. aeruginosa* through biochemical profiling. All presumptive isolates were subjected to PCR targeting the *lecB* gene for species‐level confirmation. Of these, 36 isolates were confirmed as *Pseudomonas aeruginosa* based on the presence of the expected amplicon. The remaining 78 isolates did not yield a positive amplification (287 bp) and were excluded from further analysis. Full results of the biochemical tests performed on all 36 isolates, along with the culture media and reagents used for their characterization, are provided in Supporting Information 1 and Supporting Information 2, respectively.

The *lecB* positive 36 isolates were subsequently confirmed by Sanger sequencing. This conserved locus serves as a reliable single‐locus molecular marker for *P. aeruginosa* species‐level identification and intraspecific differentiation (Sommer et al. 2016; Tielker et al. 2005). PCR amplicons were purified using the QIAquick PCR Purification Kit (QIAGEN, Germany) according to the manufacturer's protocol prior to Sanger sequencing. Sequences were queried against the NCBI GenBank database using BLAST (Altschul et al. 1990), and isolates yielding unambiguous *P. aeruginosa* matches were retained for downstream analysis. All confirmed isolates were preserved at −80°C in 20% glycerol stocks.

### 2.2 Antibiotic Susceptibility Testing

Phenotypic antimicrobial susceptibility of all 36 confirmed *P. aeruginosa* isolates was determined by the Kirby–Bauer disk diffusion method performed on Mueller–Hinton agar (Oxoid, UK), following Clinical and Laboratory Standards Institute (CLSI) M02 guidelines (Humphries et al. 2021). Eight clinically relevant antibiotics spanning multiple drug classes were tested: Aztreonam (AT, 30 μg), Meropenem (MEM, 10 μg), Cefepime (CFM, 30 μg), Gentamicin (GN, 10 μg), Tetracycline (TET, 30 μg), Tobramycin (TOB, 10 μg), Ceftazidime (CAZ, 30 μg), and Levofloxacin (LEV, 5 μg), all sourced from Oxoid (UK). Although tetracycline is not a first‐line antipseudomonal therapeutic, it was included in the panel because its resistance gene (*floR*, encoding a phenicol/tetracycline efflux pump) was a target of PCR screening, and its high environmental prevalence in Bangladeshi hospital settings (Ramatla et al. 2025) warranted phenotypic documentation. Its inclusion also enables assessment of co‐resistance burden and co‐selective pressure across the isolate collection. Bacterial suspensions were prepared from overnight cultures and adjusted to a 0.5 McFarland turbidity standard, then uniformly inoculated onto Mueller–Hinton agar plates using sterile swabs. Antibiotic disks were applied and plates were incubated at 37°C for 18–24 h, after which inhibition zone diameters were measured in millimetres. Results were interpreted as Resistant (R), Intermediate (I), or Susceptible (S) according to CLSI M100 breakpoint criteria (Clinical and Laboratory Standards Institute (CLSI) 2010). Representative disk diffusion plates are shown in Figure S6. Isolates exhibiting resistance to at least one agent in three or more antimicrobial categories were classified as multidrug‐resistant (MDR) per the definitions established by Magiorakos et al. (Magiorakos et al. 2012). Quality control was performed using *Pseudomonas aeruginosa* ATCC 27853, with zone diameters consistently falling within CLSI‐specified reference ranges throughout the study (Clinical and Laboratory Standards Institute (CLSI) 2010).

### 2.3 DNA Extraction, ARG Detection, and Mobile Genetic Elements

Genomic DNA was extracted from all 36 confirmed *P. aeruginosa* isolates using the QIAamp DNA Mini Kit (QIAGEN, Germany) according to the manufacturer's protocol. Briefly, overnight cultures were pelleted by centrifugation, resuspended in lysis buffer, and processed through the spin‐column purification steps. Extracted DNA was quantified using a spectrophotometer and stored at −20°C until use, as per standard nucleic acid preservation protocols (Adane 2022).

PCR‐based screening was performed to detect five clinically relevant antimicrobial resistance genes (ARGs): *blaSHV*, *blaOXA‐48*, *blaTEM*, *floR*, and *aac*(*6′*)*‐Ib*. Additionally, three class integrons serving as mobile genetic elements (MGEs)—*IntI1*, *IntI2*, and *IntI3*—were screened across all isolates. All primers used in this study were sourced from previously published literature and are listed in Table S2, along with their respective annealing temperatures and expected amplicon sizes (Khademi et al. 2021; Shalmashi et al. 2022; Abdelraheem et al. 2024; Teixeira et al. 2016; Stalder et al. 2012; Mobaraki et al. 2018; Chen et al. 2012). PCR reactions were carried out in a total volume of 25 μL containing 12.5 μL of 2× Master Mix, 1 μL each of forward and reverse primer (10 pmol), and 2 μL of template DNA, with nuclease‐free water added to volume. Thermal cycling conditions comprised an initial denaturation at 95°C for 5 min, followed by 35 cycles of denaturation at 95°C for 30 s, primer‐specific annealing for 30 s, and extension at 72°C for 1 min, with a final extension at 72°C for 10 min. Amplification products were resolved by electrophoresis on 1.5% agarose gels stained with 0.5 μg/mL ethidium bromide and visualized under UV transillumination. Band sizes were confirmed against a 100 bp DNA ladder. Representative gel electrophoresis images for the screened ARGs and integrons are provided in Figures S4 and S5, respectively.

### 2.4 Whole Genome Sequencing, Phylogenetic Analysis and MLST

Seven representative isolates—WPF1 (Wound Patients Ward), 3MTW (Male Toilet), 2OB (Outdoor Unit), FT (Toilet), PD (Surgical Unit), 9PUF (Pathology Unit), and FWD (Female Ward)—were selected for whole genome sequencing (WGS) based on their diverse sampling site origins and resistance profiles, to provide mechanistic resolution of the observed phenotypic resistance landscape. Genomic DNA for sequencing was extracted as described in Section 2.3, and DNA quality and quantity were assessed using a NanoDrop spectrophotometer and Qubit fluorometer prior to library preparation. Libraries were prepared using the Illumina DNA Prep Kit according to the manufacturer's protocol. Briefly, purified genomic DNA was fragmented, end‐repaired, and ligated with Illumina‐compatible adapters incorporating unique dual‐index barcodes to enable multiplexed sequencing. Library fragment size distribution and concentration were verified using a Bioanalyzer and Qubit, respectively. Sequencing was performed on the Illumina NextSeq 2000 platform using v3 600‐cycle chemistry, generating 2 × 150 bp paired‐end reads at a minimum coverage depth of 100× per isolate.

Raw sequencing reads were assessed for quality using FastQC v0.11.9 (Andrews 2010), and adapter trimming and quality filtering were performed using Trimmomatic v0.39 with default parameters, removing reads below a quality threshold of Q20 (Bolger et al. 2014). Trimmed reads were assembled de novo using SPAdes v3.15.0 with the—careful flag to minimize mismatches and indels in the final assembly (Bankevich and Nurk 2012). Assembly quality was evaluated using QUAST v5.0 (Gurevich et al. 2013), with assemblies achieving an N50 value above 50 kb and fewer than 500 contigs retained for downstream analysis. Genome completeness and contamination were assessed using CheckM v1.1.3 (Parks et al. 2015). Assembled genomes were annotated using Prokka v1.14 (Seemann 2014), and antimicrobial resistance genes were identified using ResFinder 4.0 against the ResFinder database with a minimum identity threshold of 90% and a minimum coverage of 60% (Bortolaia et al. 2020). Acquired resistance genes and chromosomally encoded resistance determinants were all captured within this pipeline. Multilocus sequence typing (MLST) was performed for all seven sequenced isolates by uploading assembled genomes to the *Pseudomonas aeruginosa* MLST database at PubMLST (https://pubmlst.org/organisms/pseudomonas‐aeruginosa). Sequence types (STs) were assigned based on allelic profiles of the seven housekeeping loci (*acsA*, *aroE*, *guaA*, *mutL*, *nuoD*, *ppsA*, *trpE*).

For phylogenetic analysis, *lecB* gene sequences obtained by Sanger sequencing from all 36 isolates were aligned together with three reference strains (PAO1, PA14, and PA7) retrieved from the NCBI GenBank database using the ClustalW multiple sequence alignment algorithm implemented in MEGA 11 (Tamura et al. 2021). The aligned sequences were used to construct a Neighbour‐Joining phylogenetic tree under the General Time Reversible model with gamma‐distributed rate variation (GTR + G). Branch support was evaluated by bootstrap analysis with 1000 replicates, and bootstrap values greater than 50% were displayed at the corresponding nodes (Saitou and Nei 1987). The phylogenetic tree was visualized and annotated using MEGA 11. No outgroup taxon was included, as the analysis was intended to assess genetic relationships among *P. aeruginosa* isolates rather than infer rooted evolutionary relationships. The reference strains PAO1, PA14, and PA7 were included to facilitate *lecB*‐based strain family classification. Following the classification framework proposed by Sommer et al. (Sommer et al. 2016), environmental isolates were assigned to either the PAO1‐like strain family (Group I) or the PA14‐like strain family (Group II) according to their clustering with the respective reference strains. Although PA7 is a genetically distinct reference strain, it was retained to represent the broader genetic diversity of *P. aeruginosa* and was not considered to define a separate *lecB* sequence‐based strain family, consistent with previous reports that place PA7 closer to the PA14‐like strain family (Sommer et al. 2016; Tielker et al. 2005).

### 2.5 Biofilm Quantification

The biofilm‐forming capacity of all 36 *P. aeruginosa* isolates was assessed using the semiquantitative crystal violet microtiter plate assay, a widely validated method for quantifying surface‐attached biofilm biomass In vitro (Stepanović et al. 2000). Briefly, overnight cultures of each isolate grown in Luria‐Bertani (LB) broth at 37°C were diluted 1:100 in fresh LB broth to obtain a standardized inoculum. A volume of 200 μL of each diluted bacterial suspension was dispensed into individual wells of a sterile 96‐well flat‐bottomed polystyrene microtiter plate (Nunc, Denmark). Uninoculated LB broth served as the negative control and was included in triplicate on every plate to establish the baseline OD cutoff value. Plates were incubated statically at 37°C for 24 h to allow biofilm development on the polystyrene surface.

Following incubation, the planktonic bacterial suspension was carefully aspirated from each well without disturbing the surface‐attached biofilm. Wells were gently washed three times with 200 μL of sterile phosphate‐buffered saline (PBS, pH 7.4) to remove non‐adherent and loosely attached cells, and plates were air‐dried at room temperature for 30 min. The remaining adherent biofilm was fixed by heating at 60°C for 1 h. Fixed biofilms were stained by adding 200 μL of 0.1% (w/v) crystal violet solution to each well and incubating at room temperature for 15 min. Excess stain was removed by washing three times with sterile distilled water, and plates were again air‐dried completely at room temperature. Bound crystal violet was solubilized by adding 200 μL of 33% glacial acetic acid to each well and incubating for 30 min at room temperature with gentle agitation. The optical density (OD) of the solubilized stain was measured at 570 nm using a microplate reader (BioTek Instruments, USA). All isolates were tested in triplicate and mean OD values were used for analysis (Stepanović et al. 2007). Representative crystal violet‐stained microtiter plate images are shown in Figure S7.

The OD cutoff value (ODcut) was defined as three standard deviations above the mean OD of the negative control wells, as described by Stepanović et al. (Sulis et al. 2020). Isolates were classified into four categories based on their mean OD values relative to ODcut: non‐biofilm former (OD≤ODcut), weak biofilm former (ODcut<OD ≤ 2× ODcut), moderate biofilm former (2× ODcut<OD ≤ 4× ODcut), and strong biofilm former (OD > 4× ODcut). This classification scheme has been widely adopted in *P. aeruginosa* biofilm studies and provides a reproducible framework for comparing biofilm‐forming capacity across isolates (Stepanović et al. 2000; Stepanović et al. 2007).

### 2.6 Statistical Analysis

All statistical analyses were conducted on the full confirmed isolate collection (*n* = 36), except for WGS‐based comparisons, which were limited to the seven sequenced isolates (*n* = 7). Prior to analysis, raw disk diffusion zone diameters (mm) and biofilm optical density (OD₅₇₀) values were recorded without transformation. Phenotypic resistance data were categorized as Resistant (R), Intermediate (I), or Susceptible (S) per CLSI M100 breakpoints before binary classification. Biofilm OD values were not normalized; outliers were not excluded, as all values were within biologically plausible ranges confirmed by triplicate measurement. The association between phenotypic antibiotic resistance and the carriage of each of the five screened ARGs was assessed using the Chi‐square test of independence, with Fisher's exact test applied in cases where expected cell frequencies fell below five, as recommended for small sample contingency table analysis (Edwards and Fischer 1925). For each gene‐antibiotic pair, isolates were categorized as either resistant or non‐resistant phenotypically, and as either gene‐positive or gene‐negative genotypically, and the resulting 2 × 2 contingency tables were analysed at a significance threshold of *α* = 0.05 (two‐sided). Associations were considered statistically significant where *p* < 0.05, and results were reported alongside their corresponding *p* values to allow transparent interpretation of the strength of phenotypic–genotypic concordance across all gene‐antibiotic combinations. Given the exploratory nature of gene‐antibiotic association testing across multiple pairings, no correction for multiple comparisons (e.g., Bonferroni or Benjamini‐Hochberg) was applied; results should therefore be interpreted as hypothesis‐generating, and findings with *p* values close to 0.05 should be considered with caution.

Major error (ME) and very major error (VME) rates were calculated for each gene–antibiotic pair following categorical agreement conventions according to Eladawy et al. ME was defined as the proportion of phenotypically susceptible isolates that were genotypically positive for the corresponding resistance gene (false resistance prediction). VME was defined as the proportion of phenotypically resistant (*R* + I) isolates that were genotypically negative for the gene (false susceptibility prediction).

The degree of association between ARG carriage and integron presence was quantified using the Phi (*φ*) coefficient, a measure of association equivalent to the Pearson correlation coefficient for binary variables, calculated from 2 × 2 contingency tables derived from the presence/absence data for each ARG–MGE pair (Cohen 2013). Phi coefficient values were interpreted as follows: *φ* < 0.10 indicating negligible association, 0.10 ≤ *φ* < 0.30 weak association, 0.30 ≤ *φ* < 0.50 moderate association, and *φ* ≥ 0.50 strong association (Cohen 2013). All statistical calculations were performed using standard functions in Microsoft Excel and SPSS Statistics v26.0 (IBM Corp., Armonk, NY, USA).

The antibiogram resistance heatmap was constructed in RStudio v2023.06 using a combination of purpose‐specific packages. The *ggplot2* package provided the core visualization framework (H. Wickham 2016), while *pheatmap* was used for annotated heatmap rendering with hierarchical clustering of isolates and antibiotics (Kolde 2019). The *RColorBrewer* package supplied the diverging colour palettes used to visually distinguish Resistant, Intermediate, and Susceptible categories across the heatmap (Neuwirth 2014). Prior to plotting, resistance data were restructured from wide to long format using the *reshape2* package (H. J. J. O. S. S. Wickham 2007), and all data manipulation, filtering, and grouping operations were performed using the *dplyr* package (Wickham et al. 2023). All remaining graphical representations including ARG prevalence charts, MGE distribution plots, biofilm classification bar charts, and Phi coefficient correlation matrices were generated using GraphPad Prism v9.0 and R v4.2.0 with the *ggplot2* package (H. Wickham 2016). The composite figure illustrating the relationship between *lecB* sequence‐based strain family classification and biofilm formation (Figure 6), comprising a strain family‐stratified biofilm OD bar chart, an overall biofilm category distribution pie chart, a strain family‐level biofilm OD box plot with Mann–Whitney U test comparison, and a per‐strain family biofilm category proportion stacked bar chart, was generated in Python v3.14 using the matplotlib (Hunter, J.D.J.C.I.S. and Engineering 2007) and seaborn (Waskom 2021) libraries. Isolates were assigned to either the PAO1‐like strain family (*n* = 28) or the PA14‐like strain family (*n* = 8) according to their clustering in the *lecB*‐based Neighbour‐Joining phylogenetic tree described in Section 2.4, following the *lecB* sequence‐based strain family classification proposed by Sommer et al. (Sommer et al. 2016). Biofilm optical density (OD) values and categorical biofilm phenotypes were subsequently compared between the two strain families. The statistical significance of differences in biofilm biomass between strain families was evaluated using the non‐parametric Mann–Whitney U test, with *p* < 0.05 considered statistically significant.

## 3 Results

### 3.1 Phylogenetic Diversity of *P. aeruginosa* Isolates

**FIGURE 2:**
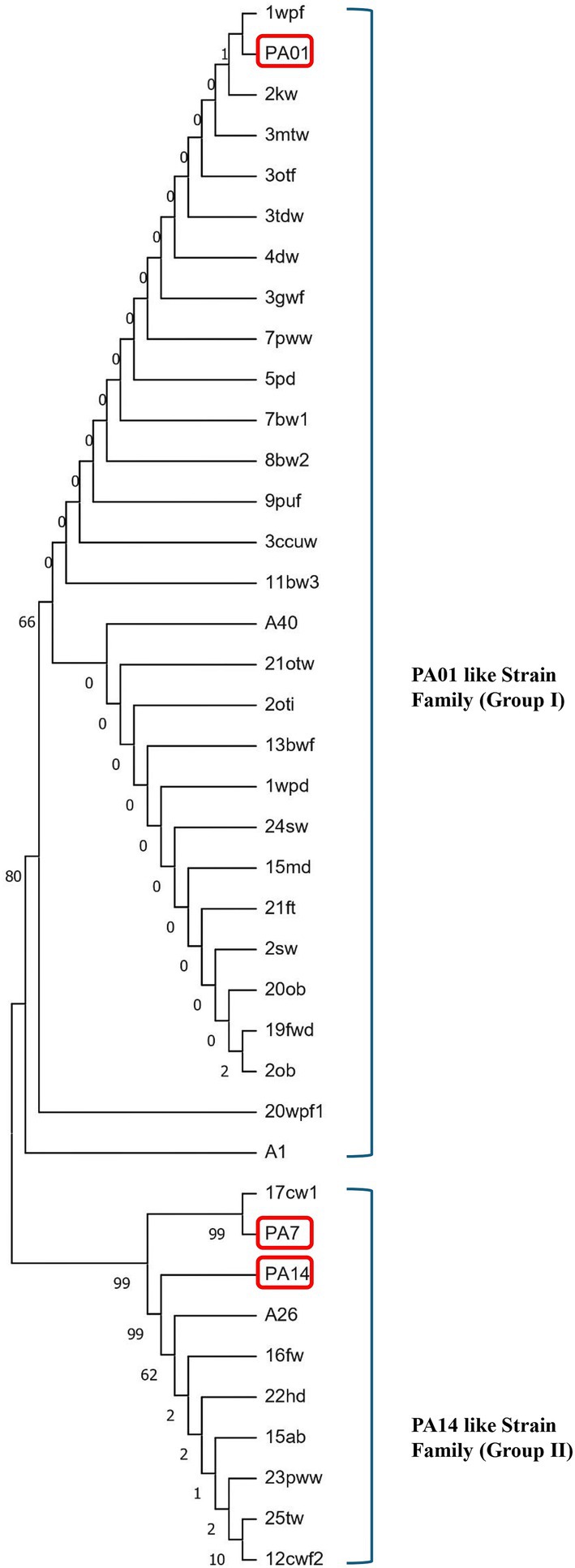
Neighbour‐Joining phylogenetic tree based on *lecB* gene sequences of 36 environmental *Pseudomonas aeruginosa* isolates and three reference strains (PAO1, PA14, and PA7). The tree was constructed in MEGA 11 using the General Time Reversible model with gamma‐distributed rate variation (GTR + G), and branch support was assessed by bootstrap analysis with 1000 replicates. Bootstrap values ≥ 41% are shown at the corresponding internal nodes. Reference strains (highlighted in red boxes) were included to facilitate *lecB*‐based strain family classification. The environmental isolates clustered predominantly into the two major *P. aeruginosa* strain families represented by PAO1 (Group I; *n* = 28) and PA14 (Group II; *n* = 8). PA7, a genetically distinct reference strain, clustered closer to the PA14‐like strain family and was included to illustrate the broader genetic diversity of *P. aeruginosa* rather than to define an additional *lecB* sequence‐based strain family. The distribution of isolates across multiple branches indicates substantial *lecB* sequence diversity within the environmental collection.

A Neighbour‐Joining phylogenetic tree was constructed using the *lecB* gene sequences of all 36 isolates together with three reference strains (PAO1, PA7, and PA14) retrieved from the NCBI GenBank database to evaluate the phylogenetic relationships among the environmental isolates and confirm their species‐level identity (Figure 2).

Phylogenetic analysis including the reference strains PAO1, PA14, and PA7 showed that the 36 environmental isolates segregated predominantly into two major *lecB* sequence groups corresponding to the PAO1‐like and PA14‐like strain families. This clustering is consistent with the established *lecB*‐based classification described by Sommer et al. (Sommer et al. 2016). Among the environmental isolates, 28 of 36 (77.8%) grouped with the PAO1‐like strain family (Group I), indicating greater sequence similarity to the PAO1 reference strain, whereas the remaining eight isolates (22.2%) clustered with the PA14‐like strain family (Group II). Although the genetically distinct reference strain PA7 was included in the analysis, it did not define an additional *lecB* sequence group among the environmental isolates. Instead, it clustered closer to the PA14‐like strains, consistent with previous reports describing its unique genomic background while illustrating the broader genetic diversity within *P. aeruginosa* (Sommer et al. 2016; Tielker et al. 2005).

The 36 environmental isolates exhibited substantial genetic diversity based on *lecB* sequences, forming multiple subgroups with generally low to moderate bootstrap support. Most internal nodes were supported by bootstrap values below 50, indicating limited confidence in the finer branching relationships, a common feature of single‐gene phylogenetic analyses of genetically diverse *P. aeruginosa* populations (Humphries et al. 2021; Clinical and Laboratory Standards Institute (CLSI) 2010). In contrast, the placement of the reference strains was strongly supported, with PA14 and PA7 resolving with bootstrap support of 100. This clustering is consistent with previous reports demonstrating that *lecB* sequence variation distinguishes the two major *P. aeruginosa* strain families, PAO1‐like and PA14‐like, while PA7 represents a genetically distinct reference strain that clusters closer to the PA14‐like group than to the PAO1‐like group (Sommer et al. 2016; Tielker et al. 2005). Consequently, the *lecB* gene is useful for assigning isolates to major strain families but provides limited resolution for inferring fine‐scale relationships among closely related isolates.

Despite the limited resolution of the *lecB*‐based phylogeny, isolates recovered from the same hospital unit were occasionally positioned in close proximity within the tree. For example, several Burn Ward isolates (8BW2, 11BW3, 13BWF, and 7BW1) and Operation Theatre isolates (2OTI, A40, 3OTF, and 21OTW) formed small local clusters, whereas isolates from other sampling locations, including the Ambulance, Pathology Unit, Gynaecology Ward, Drain, and CCU, were distributed across different branches of the tree. These observations suggest that multiple *P. aeruginosa* strain families and *lecB* sequence variants coexist within the hospital environment rather than a single dominant genotype. However, because the phylogeny is based on a single genetic locus, these clustering patterns should be interpreted cautiously and cannot be regarded as evidence of transmission, clonality, or evolutionary relatedness at the whole‐genome level. Overall, the *lecB*‐based phylogeny demonstrates substantial genetic diversity among the environmental isolates and indicates that the hospital environment harbors a heterogeneous population of *P. aeruginosa* belonging predominantly to the PAO1‐like strain family, together with a smaller proportion of PA14‐like strains.

### 3.2 Antimicrobial Resistance Profiling

**FIGURE 3:**
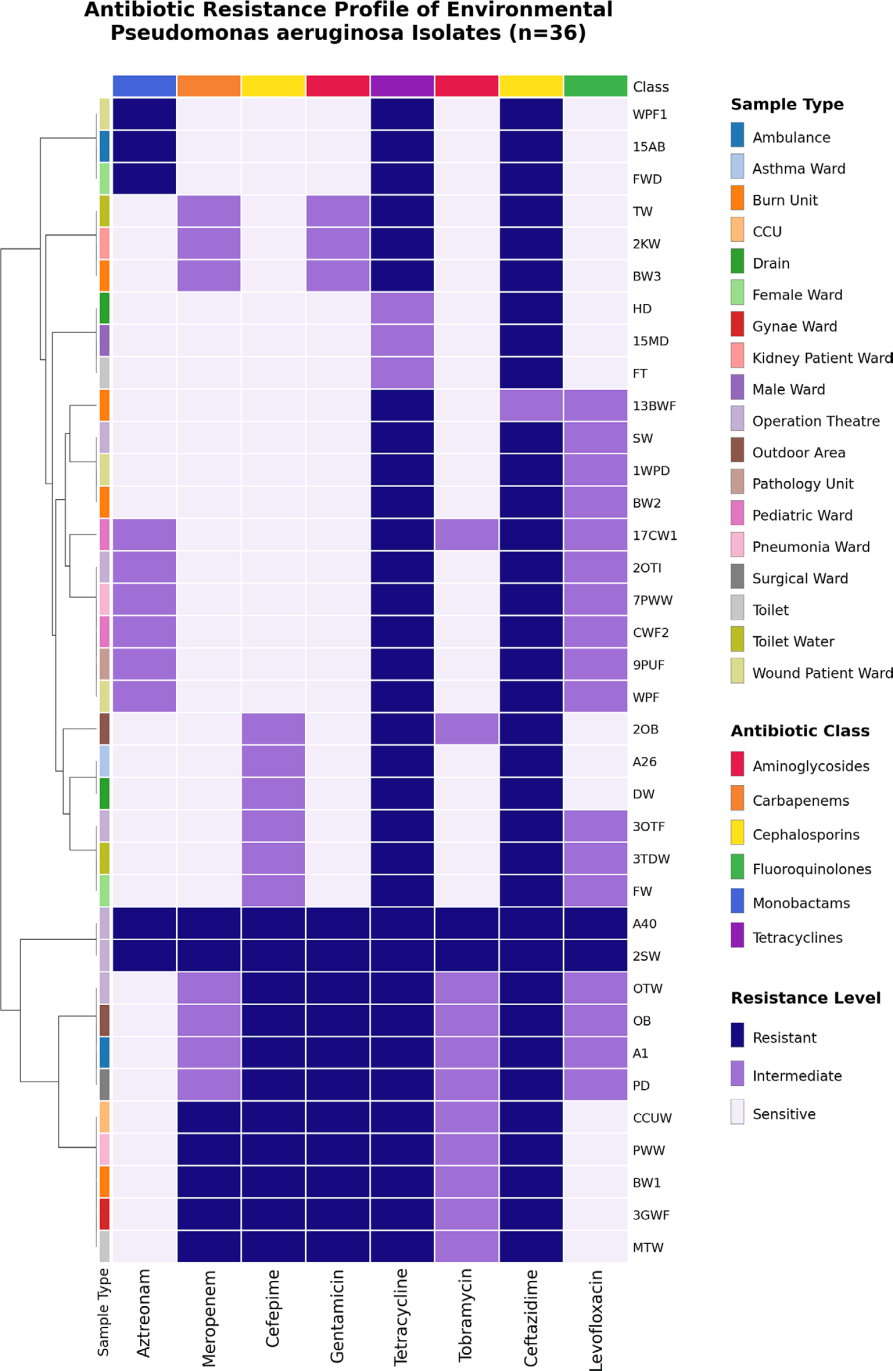
Antibiotic resistance heatmap of 36 environmental *P. aeruginosa* isolates across eight antimicrobial classes. Phenotypic antimicrobial susceptibility was determined by Kirby–Bauer disk diffusion following CLSI M100 guidelines. Each row represents an individual isolate (labelled by sample code and annotated by collection site on the left colour bar) and each column represents a tested antibiotic: Aztreonam (monobactam), meropenem (carbapenem), cefepime and ceftazidime (cephalosporins), gentamicin and tobramycin (aminoglycosides), tetracycline, and levofloxacin (fluoroquinolone). Antibiotic classes are indicated by the colour bar above the columns. Resistance levels are indicated by colour intensity: Dark blue (resistant), intermediate purple (intermediate), and white/light (sensitive), as shown in the legend. Isolates were hierarchically clustered based on their resistance profiles. *n* = 36 isolates. Resistance classification per CLSI M100 breakpoints. No statistical test was applied to the heatmap visualization; group‐level resistance rates are described in the text.

Phenotypic antimicrobial susceptibility of all 36 *P. aeruginosa* isolates was determined against eight clinically relevant antibiotics spanning multiple drug classes—Aztreonam (monobactam), Meropenem (carbapenem), Cefepime and Ceftazidime (cephalosporins), Gentamicin and Tobramycin (aminoglycosides), Tetracycline (tetracycline class), and Levofloxacin (fluoroquinolone), and the resistance profiles are presented as a heatmap (Figure 3; full isolate‐level zone diameters and CLSI interpretations in Supporting Information 3).

Ceftazidime exhibited the highest resistance rate, with 35 of 36 isolates (97.2%) classified as resistant and the remaining isolate (2.8%) as intermediate, yielding a complete absence of susceptible isolates. Tetracycline resistance was similarly pervasive, recorded in 33 isolates (91.7%), with none classified as fully susceptible. Cefepime and Gentamicin each showed resistance in 11 isolates (30.6%), followed by Meropenem in 7 isolates (19.4%). Resistance to Aztreonam was observed in 5 isolates (13.9%), while Tobramycin and Levofloxacin each exhibited the lowest resistance frequencies (5.6%, *n* = 2 each). Notably, 47.2% of isolates displayed intermediate susceptibility to Levofloxacin, indicating a clinically significant reduction in susceptibility across the collection. At the isolate level, A40 and 2SW (both from the Operation Theatre) demonstrated resistance to all eight tested antibiotics. While this profile represents resistance across all tested drug classes, formal classification as pandrug‐resistant (PDR) would require standardized testing across all clinically relevant antipseudomonal antimicrobial categories; these isolates are therefore designated as exhibiting resistance to all agents in the tested panel, consistent with an extensively drug‐resistant (XDR) phenotype. These isolates were not included in the WGS subset; sequencing was instead designed to maximize diversity of sampling site origins across the collection. This constitutes a significant limitation, as the genomic determinants underlying the most extensive resistance phenotypes in the collection—present in the Operation Theatre isolates—remain uncharacterized. Future studies should prioritize WGS of isolates exhibiting the most resistant phenotypes to enable mechanistic resolution of extreme resistance profiles. Given that A40 and 2SW were not included in the WGS subset, their resistance mechanisms cannot be directly determined from genomic data. Possible contributing determinants may include the intrinsic and acquired resistance genes identified in the sequenced isolates, as well as the ARGs and integrons detected by PCR in the full collection; however, direct inference is not warranted without sequencing of these isolates specifically.

Whole genome sequencing (WGS) of seven representative isolates—WPF1 (Wound Patients Ward), 3MTW (Male Toilet), 2OB (Outdoor Unit), FT (Toilet), PD (Surgical Unit), 9PUF (Pathology Unit), and FWD (Female Ward) revealed a repertoire of intrinsic and acquired resistance determinants within the sequenced subset (*n* = 7), providing partial mechanistic insight into the phenotypic resistance profiles observed across the collection (Table 2). The gene *aph*(*3′*)*‐IIb*, conferring aminoglycoside resistance, was identified in all sequenced isolates, as were *blaOXA‐50*, an intrinsic oxacillinase mediating resistance to Amoxicillin and Ampicillin; and the efflux‐associated genes *catB7* (chloramphenicol resistance) and *fosA* (fosfomycin resistance). *blaPAO*, conferring resistance to Amoxicillin, Ampicillin, Cefepime, and Ceftazidime, was detected in five of the seven isolates. The tetracycline resistance gene *tmexD2* was detected in six of seven isolates, consistent with the near‐universal tetracycline resistance observed phenotypically. The fluoroquinolone resistance gene *crpP* was identified in two isolates—FT (Toilet) and 9PUF (Pathology Unit), concordant with the reduced levofloxacin susceptibility noted in the disc diffusion assay.

**TABLE 2:** Antimicrobial resistance genes identified by whole genome sequencing (WGS) in seven representative *P. aeruginosa* isolates.

WPF1	3MTW	2OB	FT	PD	9PUF	FWD	Phenotypes
(*3′*)*‐IIb*	*aph*(*3′*)*‐IIb*	*aph*(*3′*)*‐IIb*	*aph*(*3′*)*‐IIb*	*aph*(*3′*)*‐IIb*	*aph*(*3′*)*‐IIb*	*aph*(*3′*)*‐IIb*	Kanamycin, Neomycin, Paromomycin, Ribostamycin, Butiromycin, Gentamicin, Unknown Aminoglycoside
*blaOXA‐50*	*blaOXA‐50*	*blaOXA‐50*	*blaOXA‐50*	*blaOXA‐50*	*blaOXA‐50*	*blaOXA‐50*	Amoxicillin, Ampicillin
*blaPAO*	*blaPAO*		*blaPAO*		*blaPAO*	*blaPAO*	Amoxicillin, Ampicillin, Cefepime, Ceftazidime
*catB7*	*catB7*	*catB7*	*catB7*	*catB7*	*catB7*	*catB7*	Chloramphenicol
*fosA*	*fosA*	*fosA*	*fosA*	*fosA*	*fosA*	*fosA*	Fosfomycin
*tmexD2*		*tmexD2*	*tmexD2*	*tmexD2*	*tmexD2*	*tmexD2*	Doxycycline, Tetracycline, Minocycline, Tigecycline
			*crpP*		*crpP*		Ciprofloxacin
MLST sequence type							
*ST12*	*ST12*	*ST12*	*ST12* *	*ST12*	*ST12*	*ST12*	

*Note:* Seven isolates—WPF1 (Wound Patients Ward), 3MTW (Male Toilet), 2OB (Outdoor Unit), FT (Toilet), PD (Surgical Unit), 9PUF (Pathology Unit), and FWD (Female Ward)—were subjected to Illumina paired‐end WGS. Resistance genes were identified using ResFinder 4.0 against the ResFinder database (minimum identity 90%, minimum coverage 60%). Each row represents a detected resistance determinant and the associated resistance phenotypes; the presence of a gene in a given isolate is indicated by the gene name in the corresponding cell, and empty cells indicate absence. Genes include intrinsic chromosomally encoded determinants (*aph*(*3′*)*‐IIb*, *blaOXA‐50*, *blaPAO*, *catB7*, *fosA*) and acquired mobile resistance genes (*tmexD2*, *crpP*).

* guaA locus was not recovered from the assembly; ST12 assignment is based on six matching loci.

### 3.3 Prevalence, Distribution, and Correlation of Antimicrobial Resistance Genes

MLST analysis of the seven sequenced isolates identified a uniform sequence type across the collection, with all isolates assigned to ST12 (allelic profile: *acsA*‐28, *aroE*‐5, *guaA*‐1, *mutL*‐3, *nuoD*‐4, *ppsA*‐4, *trpE*‐120). In isolate FT (Toilet), the *guaA* locus could not be recovered from the assembly, though all remaining six loci matched the ST12 profile. ST12 is a globally distributed lineage of *P. aeruginosa* that has been documented in clinical and environmental settings worldwide, including in association with chronic infections in immunocompromised patients.

**FIGURE 4:**
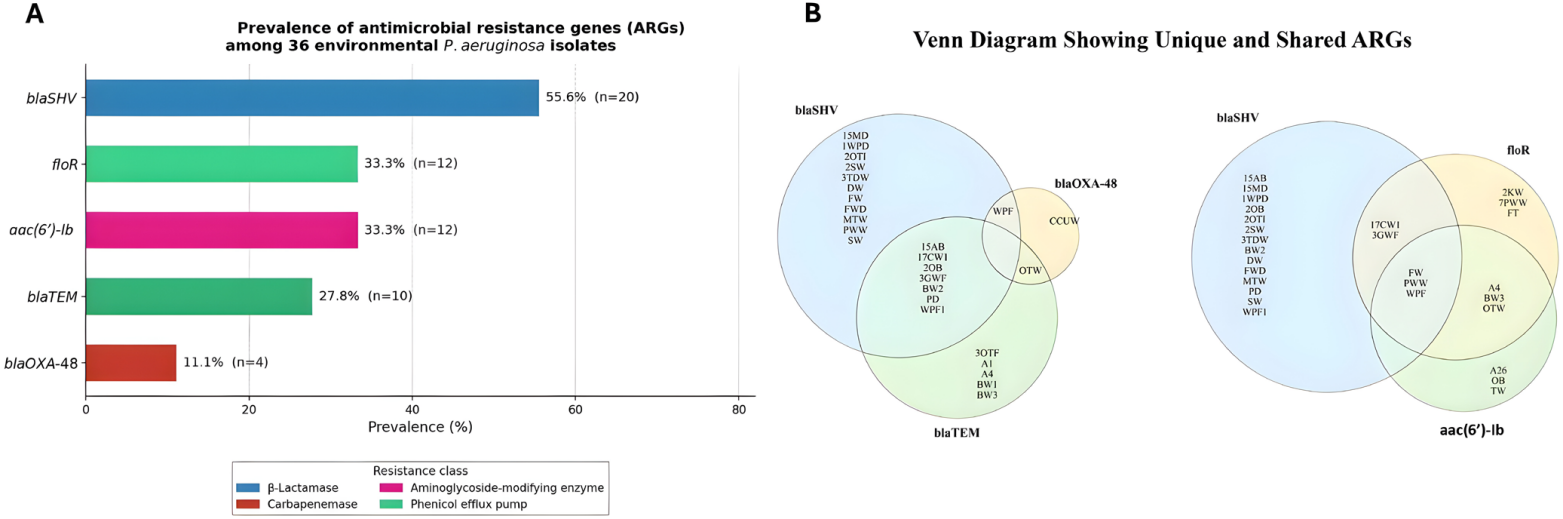
Prevalence and distribution of antimicrobial resistance genes (ARGs) among 36 environmental *P. aeruginosa* isolates. (A) Horizontal bar chart showing the prevalence (%) of five PCR‐screened ARGs: *BlaSHV* (β‐lactamase, blue), *floR* (phenicol efflux pump, green), *aac*(*6′*)*‐Ib* (aminoglycoside‐modifying enzyme, pink), *blaTEM* (β‐lactamase, green), and *blaOXA‐48* (carbapenemase, red). Numbers of positive isolates (n) are indicated at the end of each bar. (B) Venn diagrams illustrating the co‐occurrence patterns of ARGs across the 36 isolates. The left diagram shows the overlap between *blaSHV*, *blaOXA‐48*, and *blaTEM*; the right diagram shows the overlap between *blaSHV*, *floR*, and *aac*(*6′*)*‐Ib*. Individual isolate identifiers are listed within each region.

PCR‐based screening of 36 isolates for five antimicrobial resistance genes (ARGs)—*blaSHV*, *blaOXA‐48*, *blaTEM*, *floR*, and *aac*(*6′*)*‐Ib*—revealed a high overall prevalence of resistance determinants (Figure 4A; raw PCR results in Supporting Information 4). *blaSHV* was the most prevalent gene, detected in 20 isolates (55.6%), followed by *floR* and *aac*(*6′*)*‐Ib* (12 isolates each; 33.3%). *blaTEM* was present in 10 isolates (27.8%), while *blaOXA‐48* showed the lowest prevalence (4 isolates; 11.1%). Three isolates (13BWF, CWF2, and HD) were negative for all screened ARGs.

Analysis of ARG co‐occurrence patterns (Figure 4B) demonstrated substantial genetic diversity. Among β‐lactamase genes, *blaSHV* was detected alone in 11 isolates, while *blaTEM* occurred as a single gene in four isolates and *blaOXA‐48* in one isolate. Co‐carriage of *blaSHV* and *blaTEM* was observed in seven isolates, whereas limited co‐occurrence involving *blaOXA‐48* was noted. Combined presence of *blaSHV*, *floR*, and *aac*(*6′*)*‐Ib* was identified in multiple isolates, and the highest ARG burden (four genes) was observed in isolates A40, WPF, and OTW. Overall, nine isolates harboured three ARGs, indicating frequent multi‐gene carriage within the collection. Given the number of gene–antibiotic comparisons performed (40 tests), these findings should be interpreted as exploratory.

Associations between phenotypic resistance and ARG carriage were evaluated using Chi‐square analysis (*p* < 0.05) (Table 3). A significant overall association between phenotypic and genotypic resistance was observed. At the gene level, *blaSHV* was significantly associated with resistance to aztreonam, meropenem, gentamicin, and tobramycin, but not with tetracycline, cefepime, ceftazidime, or levofloxacin. *blaTEM* showed significant associations with meropenem, cefepime, gentamicin, levofloxacin, and tobramycin resistance. *floR* was significantly associated with meropenem, cefepime, levofloxacin, and tobramycin resistance, while *aac*(*6′*)*‐Ib* exhibited a comparable pattern of significant associations, concentrated in meropenem, cefepime, gentamicin, and tobramycin. Although *blaOXA‐48* showed a significant association with tobramycin resistance, this is unlikely to reflect a direct mechanistic relationship and may instead result from co‐carriage or the small number of positive isolates (*n* = 4). Notably, ceftazidime and tetracycline resistance showed no significant association with any of the screened ARGs.

**TABLE 3:** Association between phenotypic and genotypic antimicrobial resistance patterns of environmental *P. aeruginosa* isolates, with major error (ME) and very major error (VME) rates.

Antibiotic	*blaSHV* (*n* = 20)			*blaOXA‐48* (*n* = 4)			*blaTEM* (*n* = 10)			*floR* (*n* = 12)			*aac*(*6′*)*‐Ib* (*n* = 12)		
	No. (%)	ME (%)	VME (%)	No. (%)	ME (%)	VME (%)	No. (%)	ME (%)	VME (%)	No. (%)	ME (%)	VME (%)	No. (%)	ME (%)	VME (%)
Aztreonam	8 (40.0^a^)	48.0	27.3	2 (50.0^b^)	8.0	81.8	3 (30.0^b^)	28.0	72.7	4 (33.3^b^)	32.0	63.6	3 (25.0^b^)	36.0	72.7
Meropenem	6 (30.0^a^)	63.6	57.1	2 (50.0^b^)	9.1	85.7	6 (60.0^a^)	18.2	57.1	6 (50.0^a^)	27.3	57.1	7 (58.33^a^)	22.7	50.0
Cefepime	10 (50.0^b^)	52.6	41.2	2 (50.0^b^)	10.5	88.2	6 (60.0^a^)	21.1	64.7	6 (50.0^a^)	31.6	64.7	8 (66.66^a^)	21.1	52.9
Gentamicin	6 (30.0^a^)	63.6	57.1	2 (50.0^b^)	9.1	85.7	6 (60.0^a^)	18.2	57.1	6 (50.0^b^)	27.3	57.1	7 (58.33^a^)	22.7	50.0
Tetracycline	20 (100.0^b^)	NA	44.4	4 (100.0^b^)	NA	88.9	10 (100.0^b^)	NA	72.2	12 (100.0^b^)	NA	66.7	12 (100.0^b^)	NA	66.7
Tobramycin	8 (40.0^a^)	44.0	39.1	2 (50.0^a^)	8.0	81.8	5 (50.0^a^)	20.0	54.5	6 (50.0^a^)	25.0	47.8	7 (58.33^a^)	20.0	30.4
Ceftazidime	20 (100.0^b^)	NA	44.4	4 (100.0^b^)	NA	88.9	10 (100.0^b^)	NA	72.2	12 (100.0^b^)	NA	66.7	12 (100.0^b^)	NA	66.7
Levofloxacin	11 (55.0^b^)	52.9	42.1	3 (75.0^b^)	5.9	84.2	6 (60.0^a^)	23.5	68.4	6 (50.0^a^)	35.3	68.4	6 (50.0^b^)	35.3	68.4

*Note:* The table presents the number and percentage of isolates carrying each of five PCR‐detected ARGs—*blaSHV* (*n* = 20), *blaOXA‐48* (*n* = 4), *blaTEM* (*n* = 10), *floR* (*n* = 12), and *aac*(*6′*)*‐Ib* (*n* = 12)—that exhibited phenotypic resistance to each of eight tested antibiotics: Aztreonam (AT), meropenem (MEM), cefepime (CFM), gentamicin (GN), tetracycline (TET), tobramycin (TOB), ceftazidime (CAZ), and levofloxacin (LEV). For each gene–antibiotic pairing, the major error (ME) rate—the percentage of phenotypically susceptible isolates that were genotypically positive for the gene—and the very major error (VME) rate—the percentage of phenotypically resistant (*R* + I) isolates that were genotypically negative for the gene—are also reported. ME could not be calculated (NA) for tetracycline and ceftazidime, as no isolate in the collection was phenotypically susceptible to either agent. Associations between phenotypic and genotypic resistance were evaluated by Chi‐square test or Fisher's exact test at a significance threshold of *p* < 0.05. Superscript a denotes a statistically significant association; superscript b denotes a non‐significant association.

^a^ Significant association (*p* < 0.05).

^b^ Non‐significant association. ME and VME could not be calculated (NA) for tetracycline and ceftazidime because zero isolates in the collection were phenotypically susceptible to either agent.

Phenotypic–genotypic concordance was further assessed by calculating major error (ME) and very major error (VME) rates. VME rates were uniformly high (36.4%–88.9%) across most gene–antibiotic pairs, indicating that phenotypic resistance was frequently present in the absence of the corresponding gene, consistent with the involvement of additional, non‐target resistance mechanisms. ME rates were comparatively lower (5.9%–63.6%), reflecting a smaller proportion of false‐resistance prediction by gene carriage alone. For tetracycline and ceftazidime, ME could not be calculated (NA) because no isolate in the collection was phenotypically susceptible to either agent; VME for these two antibiotics ranged from 44.4% to 88.9% depending on gene prevalence. The *blaOXA‐48* gene consistently showed the highest VME rates (81.8%–88.9%) of the five genes screened, consistent with its low prevalence (*n* = 4) limiting its predictive value.

### 3.4 Mobile Genetic Elements and Their Association With Antimicrobial Resistance Genes

**FIGURE 5:**
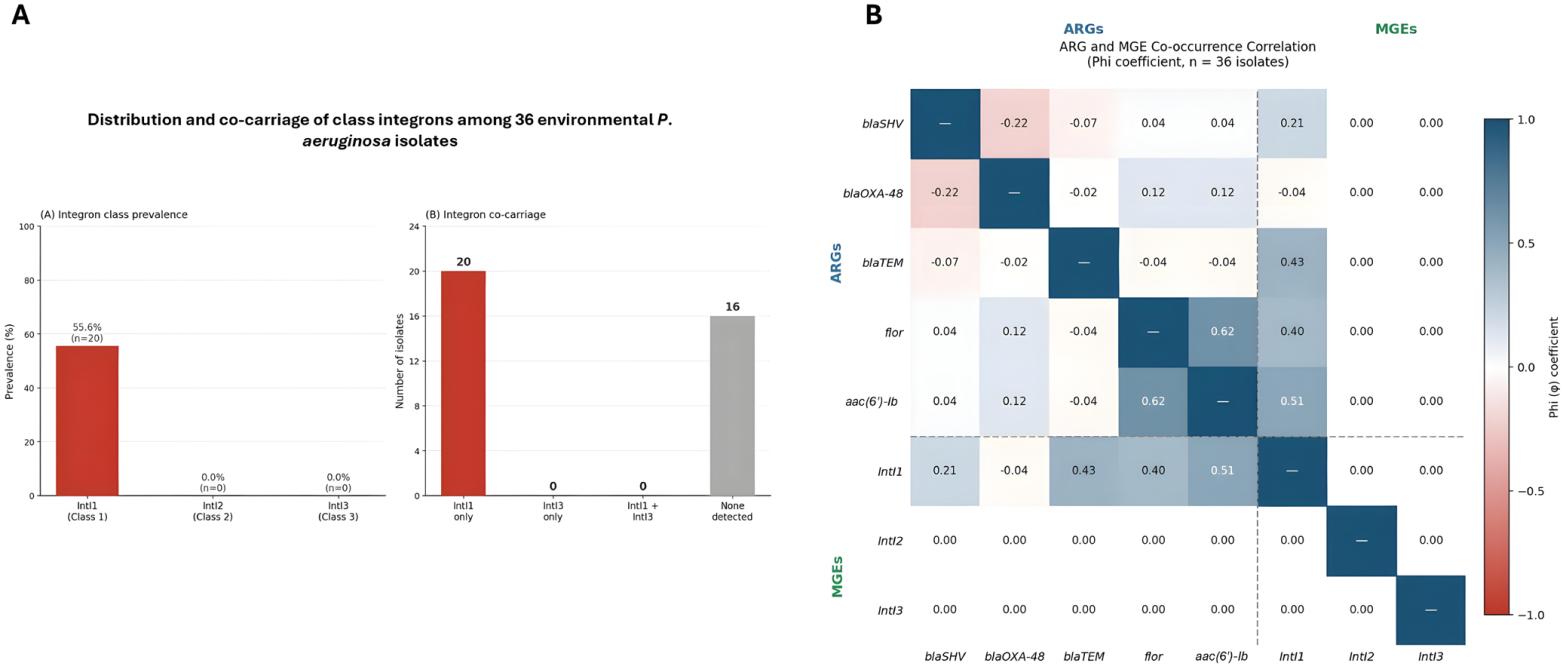
Class integron prevalence, co‐carriage patterns, and ARG–MGE co‐occurrence correlations among 36 environmental *P. aeruginosa* isolates. (A, left panel) Bar chart showing the prevalence (%) of class 1 (*IntI1*), class 2 (*IntI2*), and class 3 (*IntI3*) integrons across the collection. *IntI1* was detected in 55.6% (*n* = 20) of isolates; *IntI2* and *IntI3* were absent (0.0%). (A, right panel) Bar chart showing integron co‐carriage patterns: 20 isolates carried *IntI1* only, while 16 harboured no detectable integron; no co‐carriage of multiple integron classes was observed. (B) Phi (*φ*) coefficient correlation matrix depicting pairwise co‐occurrence associations between five ARGs (*blaSHV*, *blaOXA‐48*, *blaTEM*, *floR*, *aac*(*6′*)*‐Ib*) and three MGE classes (*IntI1*, *IntI2*, *IntI3*) across the 36 isolates. Colour intensity and direction (dark blue = positive; red = negative) reflect the strength and direction of association, with *φ* values displayed within each cell. The dashed lines demarcate ARG–ARG, ARG–MGE, and MGE–MGE correlation quadrants. Phi (*φ*) coefficient values were calculated from 2 × 2 contingency tables of presence/absence data for each ARG–MGE pair across *n* = 36 isolates. No formal *p* value threshold was applied to the *φ* coefficient; values are reported as continuous measures of association strength.

*IntI1* was the sole integron class detected in this collection, identified in 20 isolates (55.6%). Both *IntI2* and *IntI3* were absent from all 36 isolates (0%), indicating their non‐involvement in resistance gene mobilization within this hospital environment. The remaining 16 isolates (44.4%) harboured no detectable integron, and all *IntI1*‐negative isolates corresponded entirely to single‐integron‐class carriage, with no co‐carriage events observed. All 20 *IntI1*‐positive isolates harboured the integron as their sole detected MGE class (Figure 5A; raw MGE screening results in Supporting Information 4).

The strongest association observed in the entire matrix was between *floR* and *aac*(*6′*)*‐Ib* (*φ* = 0.62), reflecting highly structured co‐occurrence of the phenicol efflux and aminoglycoside resistance determinants within the collection (Figure 5B). Among ARG–MGE associations, *floR* demonstrated the strongest correlation with *IntI1* (*φ* = 0.40), suggesting a moderate linkage between *floR* carriage and class 1 integron presence, though causal inference from correlation data alone warrants caution. *aac*(*6′*)*‐Ib* similarly showed a moderate positive association with *IntI1* (*φ* = 0.51), implicating integron‐mediated mobility in the dissemination of this aminoglycoside resistance gene. *blaTEM* exhibited a moderate positive correlation with *IntI1* (*φ* = 0.43), while *blaSHV* showed a weak positive association (*φ* = 0.21). *blaOXA‐48* demonstrated a negligible association with *IntI1* (*φ* = −0.04), suggesting its dissemination is predominantly mediated through integron‐independent mobile elements such as plasmids or transposons. Across all pairings, both *IntI2* and *IntI3* exhibited zero correlation with every ARG assessed (*φ* = 0.00 throughout), consistent with their complete absence from the collection and confirming their irrelevance as resistance gene vehicles in this setting.

### 3.5 Biofilm‐Forming Capacity of Isolates

The biofilm‐forming capacity of all 36 *P. aeruginosa* isolates was quantified by crystal violet microtiter plate assay, with isolates classified as strong, moderate, weak, or non‐biofilm formers based on optical density (OD) values relative to the OD cutoff threshold (ODcut = 0.065) (Table 4).

**TABLE 4:** Biofilm‐forming capacity of 36 environmental *P. aeruginosa* isolates determined by crystal violet microtiter plate assay.

SampleID	Mean	SD	OD (Mean + SD)	ODcut	Biofilm property
1WPD	0.0700	0.07086	0.14165	0.065	Moderate‐biofilm‐former
A40	0.23517	0.03086	0.26602		Strong biofilm‐former
15AB	0.12037	0.01490	0.13527		Moderate biofilm‐former
2KW	0.13220	0.02159	0.15379		Moderate biofilm‐former
13BWF	0.10890	0.03275	0.14165		Moderate biofilm‐former
A1	0.17097	0.05196	0.22293		Strong biofilm‐former
9PUF	0.29487	0.05307	0.34794		Strong biofilm‐former
17CW1	0.15380	0.02430	0.17810		Strong biofilm‐former
3GWF	0.06380	0.01095	0.07475		Weak biofilm‐former
A26	0.14437	0.00646	0.15083		Moderate biofilm‐former
3TDW	0.08413	0.01862	0.10275		Moderate biofilm‐former
15MD	0.06170	0.00606	0.06776		Moderate‐biofilm‐former
WPF	0.09520	0.01230	0.10750		Moderate biofilm‐former
MTW	0.21045	0.02560	0.23605		Strong biofilm‐former
DW	0.07080	0.00890	0.07970		Weak biofilm‐former
PD	0.16020	0.02010	0.18030		Strong biofilm‐former
FWD	0.11850	0.01540	0.13390		Moderate biofilm‐former
OB	0.05560	0.0710	0.1266		Moderate‐biofilm‐former
FT	0.24530	0.02870	0.27400		Strong biofilm‐former
BW1	0.13540	0.01980	0.15520		Moderate biofilm‐former
BW2	0.09010	0.01020	0.10030		Moderate biofilm‐former
BW3	0.06240	0.00310	0.06550		Non‐biofilm‐former
CWF2	0.17890	0.02250	0.20140		Strong biofilm‐former
FW	0.14220	0.01870	0.16090		Moderate biofilm‐former
WPF1	0.08050	0.00960	0.09010		Weak biofilm‐former
OTW	0.23040	0.03120	0.26160		Strong biofilm‐former
HD	0.12570	0.01740	0.14310		Moderate biofilm‐former
PWW	0.06680	0.00420	0.07100		Weak biofilm‐former
SW	0.15560	0.02080	0.17640		Strong biofilm‐former
TW	0.11230	0.01390	0.12620		Moderate biofilm‐former
7PWW	0.05420	0.0780	0.1322		Moderate‐biofilm‐former
3OTF	0.19840	0.02750	0.22590		Strong biofilm‐former
2OTI	0.13950	0.01680	0.15630		Moderate biofilm‐former
CCUW	0.07340	0.00910	0.08250		Weak biofilm‐former
2OB	0.26070	0.03540	0.29610		Strong biofilm‐former
2SW	0.08050	0.00960	0.09010		Weak biofilm‐former

*Note:* Biofilm formation was quantified by measuring the optical density at 570 nm (OD_570_) of crystal violet‐stained surface‐attached biomass following 24‐h static incubation at 37°C. Values presented are the mean OD, standard deviation (SD), and mean + SD for each isolate, each determined from triplicate measurements. The OD cutoff value (OD cut = 0.065), defined as three standard deviations above the mean OD of uninoculated negative control wells, was used to classify isolates into four biofilm categories: Non‐biofilm former (OD≤OD cut), weak biofilm former (OD cut<OD ≤ 2× OD cut), moderate biofilm former (2× OD cut<OD ≤ 4× OD cut), and strong biofilm former (OD > 4× OD cut), following the classification scheme of Stepanović et al.

Strong biofilm formation was exhibited by 12 isolates (33.3%), with 9PUF (Pathology Unit) recording the highest OD value across the collection (OD = 0.3479), followed by 2OB (Outdoor Unit, OD = 0.2961), FT (Toilet, OD = 0.2740), A40 (Operation Theatre, OD = 0.2660), and OTW (Operation Theatre, OD = 0.2616). Additional strong biofilm formers included MTW (Male Toilet), 3OTF (Operation Theatre), A1 (Ambulance), CWF2 (Paediatric Ward), PD (Surgical Unit), 17CW1 (Paediatric Ward), and SW (Operation Theatre). Moderate biofilm formation, the most prevalent category, was recorded in 17 isolates (47.2%) from a diverse range of sampling sites including the Outdoor Unit, Female Ward, Operation Theatre, Burn Unit, Kidney Ward, Asthma Ward, Drain, Wound Patients Ward, Ambulance, Toilet Water, and Pneumonia Ward. Weak biofilm formation was observed in 6 isolates (16.7%)– WPF1 (Wound Patients Ward), 2SW (Operation Theatre), CCUW (CCU), DW (Drain), 3GWF (Gynaecology Ward), and PWW (Pneumonia Ward), each with OD values near the cutoff threshold. Only one isolate, BW3 (Burn Unit, OD = 0.0655), was classified as a non‐biofilm former.

Overall, 97.2% of isolates (35/36) demonstrated measurable biofilm‐forming capacity, with 80.5% (29/36) classified as moderate to strong formers. Notably, several MDR isolates from the Operation Theatre, including A40, OTW, and 2SW, also exhibited strong or moderate biofilm formation, indicating a potential convergence of antimicrobial resistance and biofilm‐mediated persistence within this high‐risk clinical environment.

### 3.6 Correlation of *lecB* Sequence‐Based Phylogenetic Clustering With Biofilm Formation

**FIGURE 6:**
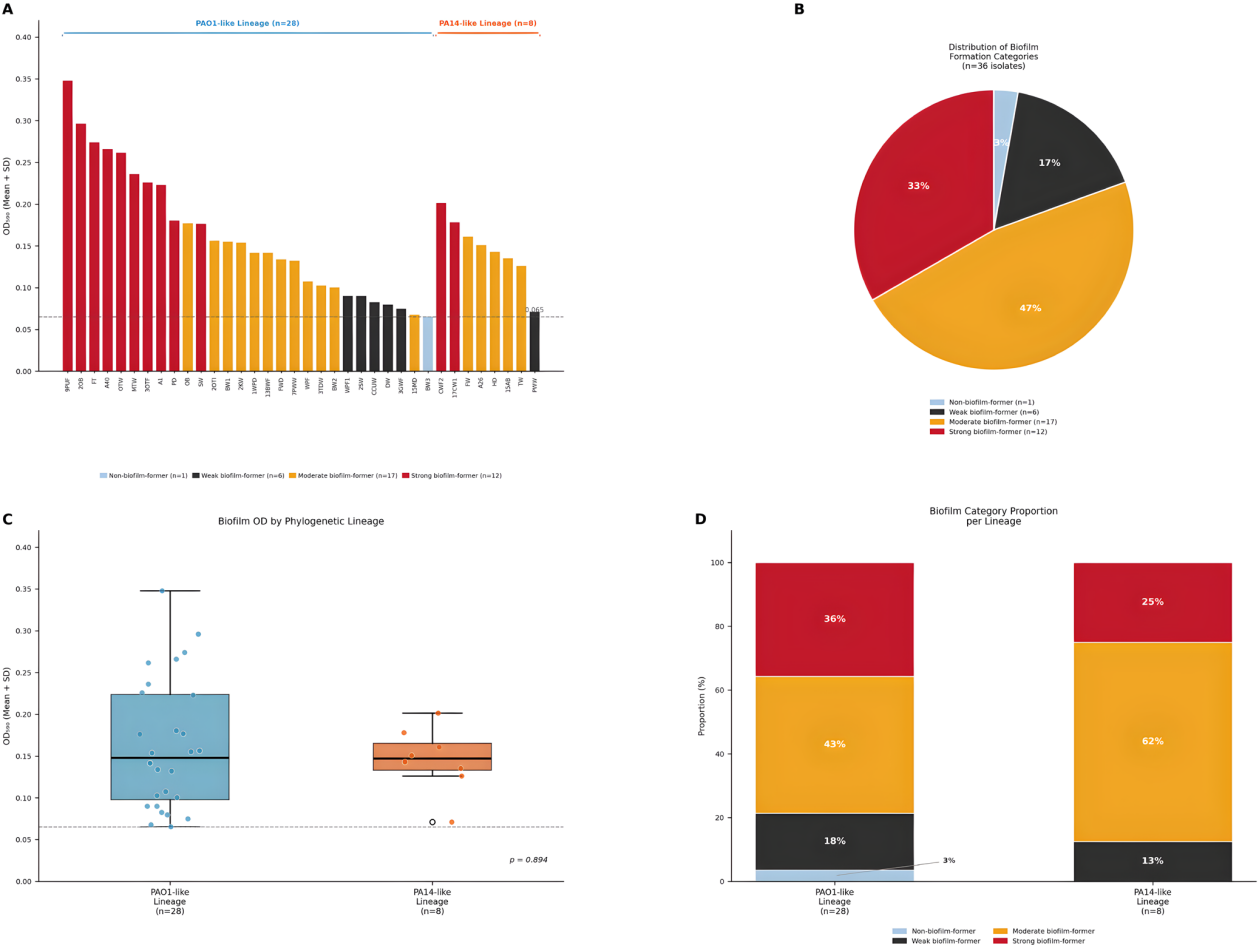
Correlation between *lecB* sequence‐based strain family classification and biofilm formation in environmental *Pseudomonas aeruginosa* isolates (*n* = 36). (A) Bar chart showing biofilm optical density (OD570; mean ± SD) of all 36 isolates grouped according to *lecB*‐based strain family classification into the PAO1‐like strain family (Group I, *n* = 28) and the PA14‐like strain family (Group II, *n* = 8). The horizontal dashed line indicates the biofilm cutoff value (ODcut = 0.065). Bars are coloured according to biofilm phenotype: Strong (red), moderate (orange), weak (black), and non‐biofilm former (light blue). (B) Overall distribution of biofilm phenotypes among the 36 isolates: Strong (33.3%, *n* = 12), moderate (47.2%, *n* = 17), weak (16.7%, *n* = 6), and non‐biofilm former (2.8%, *n* = 1). (C) Box plot comparing biofilm OD values between the PAO1‐like and PA14‐like strain families, with individual isolates overlaid. The horizontal dashed line indicates ODcut = 0.065. Differences between strain families were evaluated using the Mann–Whitney U test (*p* = 0.894). (D) Distribution of biofilm phenotypes within each *lecB*‐based strain family. Among PAO1‐like isolates (*n* = 28), 36% were strong, 43% moderate, 18% weak, and 3% non‐biofilm formers, whereas PA14‐like isolates (*n* = 8) comprised 25% strong, 62% moderate, and 13% weak biofilm formers. Biofilm OD values represent the mean ± SD of triplicate measurements. The biofilm cutoff (ODcut = 0.065) was defined as the mean OD of the negative control plus three standard deviations.

To investigate the relationship between *lecB* sequence‐based strain family classification and biofilm formation, all 36 isolates were assigned to either the PAO1‐like strain family (*n* = 28) or the PA14‐like strain family (*n* = 8) based on the *lecB*‐based Neighbour‐Joining phylogeny, and their biofilm optical density (OD) values and categorical biofilm phenotypes were compared (Figure 6).

The *lecB*‐based phylogenetic analysis assigned 28 isolates (77.8%) to the PAO1‐like strain family (Group I) and eight isolates (22.2%) to the PA14‐like strain family (Group II) (Figure 6A). Overall, 35 of the 36 isolates (97.2%) produced biofilms with OD values exceeding the calculated cutoff (ODcut = 0.065). Based on the established classification criteria, 12 isolates (33.3%) were classified as strong biofilm formers, 17 (47.2%) as moderate biofilm formers, six (16.7%) as weak biofilm formers, and one isolate (2.8%) as a non‐biofilm former (Figure 6B).

Comparison of biofilm OD values between the two strain families showed that isolates belonging to the PAO1‐like strain family exhibited a broader range of biofilm production, including the highest OD values observed in the collection, whereas isolates assigned to the PA14‐like strain family displayed a narrower distribution and a lower median biofilm OD (Figure 6C). However, the difference in biofilm biomass between the two groups was not statistically significant (Mann–Whitney U test, *p* = 0.894).

Analysis of biofilm category distribution showed differences in the proportion of biofilm phenotypes between the two strain families (Figure 6D). Among the PAO1‐like strain family isolates (*n* = 28), 36% were classified as strong biofilm formers, 43% as moderate biofilm formers, 18% as weak biofilm formers, and one isolate (3%; BW3 from the Burn Unit) was identified as a non‐biofilm former. In contrast, isolates belonging to the PA14‐like strain family (*n* = 8) were predominantly moderate biofilm formers (62%), whereas strong biofilm formers accounted for 25% and weak biofilm formers for 13%, with no non‐biofilm formers detected. Overall, strong biofilm formers were proportionally more frequent in the PAO1‐like strain family (36%) than in the PA14‐like strain family (25%).

## 4 Discussion

*Pseudomonas aeruginosa* is a formidable nosocomial pathogen whose capacity to persist within hospital environments is underpinned by rapid resistance acquisition, integron‐mediated gene dissemination, and prolific biofilm formation. The findings of this study demonstrate that environmental isolates recovered across diverse hospital microenvironments harbour resistance profiles that mirror and in several respects exceed those documented in clinical collections, underscoring the hospital environment as an active reservoir shaping the local resistance landscape.

### 4.1 Phylogenetic Heterogeneity and *lecB* ‐Based Strain Family Classification of Environmental *P. aeruginosa* Isolates

The *lecB* gene encodes the fucose‐binding lectin LecB, an important virulence factor involved in bacterial adhesion and biofilm formation. Previous studies have shown that variation in the *lecB* sequence provides a useful genetic marker for classifying *P. aeruginosa* isolates into the two major strain families, PAO1‐like and PA14‐like, with the observed sequence variation closely reflecting broader genomic differences between these strain families (Sommer et al. 2016; Tielker et al. 2005). In the present study, all 36 environmental isolates clustered within the *P. aeruginosa* reference group, providing molecular confirmation of species identity and demonstrating the utility of *lecB* sequence analysis for strain family assignment.

The Neighbour‐Joining phylogeny resolved the environmental isolates predominantly into the PAO1‐like strain family (Group I) and the PA14‐like strain family (Group II), consistent with the *lecB*‐based classification framework proposed by Sommer et al. (Sommer et al. 2016). Importantly, PA7 did not define an additional *lecB* sequence group among the environmental isolates, supporting the predominance of the two principal *lecB* sequence‐based strain families described previously. Most internal branches of the phylogenetic tree were supported by relatively low bootstrap values, indicating limited confidence in the finer branching relationships among individual isolates. Such a pattern is expected when phylogenetic inference is based on a single genetic locus, which generally provides sufficient resolution for broad strain family classification but limited discriminatory power for resolving relationships among closely related isolates. Accordingly, the present phylogeny should be interpreted primarily as evidence of *lecB* sequence diversity and strain family assignment rather than as a reconstruction of the detailed evolutionary history of the environmental isolates.

Although several isolates recovered from the same hospital units, particularly the Burn Ward and Operation Theatre, occupied nearby positions within the phylogenetic tree, these local groupings were supported by relatively low bootstrap values and therefore should be interpreted cautiously. Likewise, isolates recovered from different hospital locations, including the Drain, CCU, Ambulance, Gynaecology Ward, and Pathology Unit, were distributed across multiple branches of the tree, indicating that multiple *lecB* sequence variants are present throughout the hospital environment rather than a single dominant strain family. Because the phylogeny was reconstructed from a single virulence‐associated gene, these spatial patterns should not be interpreted as evidence of clonal transmission, common environmental reservoirs, or independent colonization events. Confirmation of such epidemiological relationships would require higher‐resolution approaches such as multilocus sequence typing or whole‐genome sequencing.

Overall, the *lecB*‐based phylogenetic analysis demonstrates substantial genetic heterogeneity among environmental *P. aeruginosa* isolates and confirms that both PAO1‐like and PA14‐like strain families circulate within the hospital environment, with the PAO1‐like strain family predominating in the present collection. These findings extend previous observations on *lecB* sequence diversity and provide a framework for examining whether strain family classification is associated with clinically relevant phenotypes, including biofilm formation and antimicrobial resistance.

### 4.2 Broad‐Spectrum Phenotypic Resistance and Genomic Resistance Determinants Underscore the MDR Threat of Environmental *P. aeruginosa*

The resistance rates documented in this study exceed those previously reported from clinical and environmental *P. aeruginosa* collections in Bangladesh and neighbouring regions. A systematic review of *P. aeruginosa* resistance in Bangladesh (2006–2024) reported ceftazidime resistance in 76.2% of isolates (Ramatla et al. 2025), while studies from Dhaka Medical College Hospital documented 86.8% (Rashid et al. 2007), and a regional clinical surveillance study reported 84.6% among carbapenem‐resistant *P. aeruginosa* (Ferdus et al. 2025). The higher rates recorded in the present environmental collection suggest that sustained antibiotic selection pressure within hospital premises drives resistance escalation in the surrounding microenvironment beyond what clinical isolate surveillance alone captures, consistent with global trends of cephalosporin resistance driven by ESBL production and AmpC overexpression (Botelho et al. 2019; Poole 2011). The progressive rise in carbapenem‐resistant *P. aeruginosa* in Bangladeshi hospitals, increasingly attributed to metallo‐β‐lactamase production and *OprD* porin loss (Ramatla et al. 2025; Saha et al. 2022), contextualizes the meropenem resistance observed here as part of a broader regional resistance trajectory. The substantial intermediate susceptibility to levofloxacin warrants clinical concern, as intermediate strains may fail therapy under standard dosing regimens and serve as stepping stones toward high‐level resistance (Poole 2011). The emergence of isolates resistant to all eight tested antibiotics in the Operation Theatre is of significant concern, as extensively drug‐resistant (XDR) and pandrug‐resistant *P. aeruginosa* are associated with severely restricted therapeutic options and increased patient mortality (Botelho et al. 2019; Nathwani et al. 2014). The universal detection of *blaOXA‐50*, *blaPAO*, *fosA*, *catB7*, and *aph*(*3′*)*‐IIb* across all seven sequenced isolates is consistent with their established status as core‐genome‐encoded intrinsic resistance determinants in *P. aeruginosa* (Ahmed and Resistance 2022; Stover et al. 2000; Serepa‐Dlamini et al. 2025; Salem et al. 2024; Zaidi et al. 2023).

The *tmexCD‐toprJ* operon, originally identified in *P. aeruginosa* and increasingly detected across diverse Gram‐negative pathogens on conjugative plasmids, poses a significant public health threat given its potential for inter‐species horizontal dissemination (Lv et al. 2020; Zhu et al. 2023). Hospital surface and wastewater environments have been implicated as reservoirs sustaining the environmental persistence and onward transmission of this mobile efflux system (Zhu et al. 2023), making its detection in the present collection particularly noteworthy. *crpP*, originally identified on the conjugative plasmid pUM505, encodes a ciprofloxacin‐modifying enzyme conferring reduced fluoroquinolone susceptibility through ATP‐dependent phosphorylation (Chávez‐Jacobo et al. 2018). Subsequent studies have demonstrated widespread dissemination of *crpP*‐like genes via integrative and conjugative elements (ICEs), with detection rates as high as 61.9% among complete *P. aeruginosa* genomes (Ortiz de la Rosa et al. 2020; Botelho et al. 2020), underscoring its potential for rapid interstrain and interspecies spread within hospital environments.

A notable limitation is that the most phenotypically resistant isolates (A40 and 2SW, Operation Theatre) were not included in the WGS subset, as sequencing prioritized breadth of sampling site diversity over phenotypic extremes; the genomic basis of their resistance therefore remains uncharacterized in this study.

### 4.3 Multifaceted ARG Profiles co‐Carriage Complexity, and Phenotypic–Genotypic Discordance in Environmental *P. aeruginosa*

The predominance of *blaSHV* among the screened ARGs surpasses rates previously reported in clinical *P. aeruginosa* collections from comparable settings, ranging from 6.7% to 11.1% in Iran to 69.5% in South Africa (Teixeira et al. 2016; Fattahi et al. 2015; Hosu et al. 2021), reflecting considerable regional variation in *blaSHV* dissemination, the specific drivers of which—including antibiotic selection pressure, clonal dynamics, and gene transfer frequencies—have not been directly assessed in the present study and remain to be determined. The elevated prevalence in the present Bangladeshi hospital environment likely reflects intensified broad‐spectrum β‐lactam use, where empirical prescribing and suboptimal antibiotic stewardship amplify selective pressure for ESBL‐producing strains (Poole 2011; Ramatla et al. 2025). The *blaTEM* prevalence aligns with rates reported from clinical *P. aeruginosa* in Tehran (26.7%) (Fattahi et al. 2015) and falls within the range documented globally across diverse hospital environments (Shalmashi et al. 2022; Panahi et al. 2020). The detection of *blaOXA‐48* in environmental *P. aeruginosa* from high‐risk units is of significant epidemiological concern, as its presence is increasingly attributed to horizontal acquisition from co‐colonizing Enterobacterales via mobile genetic elements, implicating the hospital environment as a conduit for inter‐species carbapenemase gene transfer (Abdelraheem et al. 2024; El Sharief et al. 2025).

While *floR* is principally characterized as a phenicol resistance determinant, its consistent co‐occurrence with *blaSHV*, *aac*(*6′*)*‐Ib*, and integron‐associated MGEs suggests it functions as a co‐selected marker within broader resistance gene clusters, consistent with findings by Chen et al. demonstrating *floR* co‐distribution with β‐lactamase and aminoglycoside resistance genes in MDR Gram‐negative hospital isolates (Botelho et al. 2020). The *aac*(*6′*) family constitutes the most frequently detected aminoglycoside‐modifying enzyme class in *P. aeruginosa* globally, conferring resistance to gentamicin, tobramycin, and related compounds through enzymatic acetylation (Teixeira et al. 2016; Panahi et al. 2020). The co‐carriage of *blaSHV* and *blaOXA‐48* within the same isolate is of particular clinical significance, as this combination confers simultaneous ESBL and carbapenemase activity, eliminating both third‐generation cephalosporins and carbapenems as viable therapeutic options (El Sharief et al. 2025; Bush and Bradford 2016). High‐risk microenvironments subject to intensive antibiotic use are well established as the strongest drivers of such multi‐gene resistance accumulation through sustained co‐selective pressure (Botelho et al. 2019; Polse et al. 2023).

The statistically significant concordance between phenotypic and genotypic resistance patterns is consistent with prior studies reporting broad but imperfect agreement between PCR‐detected ARGs and disc diffusion phenotypes in *P. aeruginosa* (Jhang et al. 2026; Aloush et al. 2006). The uniformly high VME rates observed across most gene–antibiotic pairs (36.4%–88.9%) indicate that absence of the screened ARGs frequently failed to predict phenotypic susceptibility, underscoring the limited negative predictive value of this five‐gene panel for clinical or surveillance decision‐making. This pattern was most pronounced for *blaOXA‐48* (VME 81.8%–88.9%), consistent with its low prevalence in this collection (*n* = 4) constraining its discriminatory power. Comparatively lower ME rates (5.9%–63.6%) suggest gene carriage was a more reliable indicator of resistance than gene absence was of susceptibility, a pattern consistent with the polygenic and often redundant nature of *P. aeruginosa* resistance mechanisms, where multiple chromosomal and acquired pathways can independently produce the same resistant phenotype.

The absence of significant genotypic correlation with ceftazidime and tetracycline resistance across all five screened ARGs, despite near‐universal phenotypic resistance, reflects the primacy of chromosomally mediated mechanisms in *P. aeruginosa* ceftazidime resistance: *AmpC* β‐lactamase derepression and *OprD* porin loss are mutation‐driven events not detectable by conventional ARG screening (Lister et al. 2009; Mesaros et al. 2007; Cabot et al. 2012), highlighting the need to integrate chromosomal mutation analysis alongside PCR‐based methods for comprehensive resistance profiling of environmental collections. The ARG panel employed in this study, while covering five clinically relevant acquired resistance genes, does not encompass the full spectrum of *P. aeruginosa* resistance determinants. Important mechanisms not assessed include carbapenemases beyond *blaOXA‐48* (such as *blaNDM*, *blaVIM*, *blaIMP*, and *blaKPC*), extended‐spectrum β‐lactamases beyond *blaSHV* and *blaTEM* (particularly *blaCTX‐M* and *blaPER*), efflux pump overexpression (*mexAB‐oprM*, *mexCD‐oprJ*, *mexXY‐oprM*), *OprD* porin loss, and fluoroquinolone resistance‐determining region (QRDR) mutations. Future studies incorporating whole‐genome sequencing of all isolates or expanded multiplex PCR panels would provide a more complete resistance gene inventory.

### 4.4 Integron‐Mediated Horizontal Gene Transfer as a Dominant Driver of Resistance Gene Dissemination in Hospital Environmental *P. aeruginosa*

The *IntI1* prevalence of 55.6% documented in this study falls within the range reported from clinical *P. aeruginosa* collections worldwide, where class 1 integron carriage ranges from 37% to 95% across diverse hospital settings (Khademi et al. 2021; Yousefi et al. 2010; Ferdosi 2013; Shojapour et al. 2019), corroborating the well‐established status of *IntI1* as the most clinically prevalent and epidemiologically significant integron class in *P. aeruginosa* (Saitou and Nei 1987). The strong association between *IntI1* carriage and MDR phenotypes documented in multiple prior studies (Khademi et al. 2021; Yousefi et al. 2010; Ferdosi 2013) further contextualizes the resistance burden observed in the present collection.

The complete absence of both *IntI2* and *IntI3* across all 36 isolates is fully concordant with the broader published literature. The negligible prevalence of *IntI2* in *P. aeruginosa* is well established (Khademi et al. 2021; Faghri et al. 2018), consistent with the recognized tropism of class 2 integrons for Enterobacteriaceae rather than non‐fermenters (White et al. 2001). Similarly, class 3 integrons are reported as absent or detected only sporadically in *P. aeruginosa* in the majority of studies (Khademi et al. 2021; Shojapour et al. 2019), with rare exceptions documented at 15% in northwest Iran (Mobaraki et al. 2018) and in isolated case reports from Japan and Brazil (Zarei‐Yazdeli et al. 2018). The present findings therefore reflect the expected integron class distribution for environmental *P. aeruginosa* and contrast with the previous version of this dataset, in which *IntI3* detection was observed; the updated data confirm that class 1 integrons constitute the sole integron‐mediated resistance gene transfer vehicle operative in this collection.

The moderate *floR*–*IntI1* association (*φ* = 0.40) is mechanistically consistent with the well‐documented incorporation of *floR* within multi‐cassette integron arrays in MDR Gram‐negative bacteria, where it functions as a co‐selected resistance gene under sustained antibiotic pressure (Stalder et al. 2012; Partridge et al. 2009). The strong association of *aac*(*6′*)*‐Ib* with *IntI1* (*φ* = 0.51) is similarly consistent, as aminoglycoside resistance gene cassettes rank among the most frequently identified cassettes within class 1 integrons in *P. aeruginosa* globally (Teixeira et al. 2016; Mobaraki et al. 2018). The moderate association of *blaTEM* with *IntI1* (*φ* = 0.43) suggests partial integron‐mediated dissemination of this broad‐spectrum β‐lactamase, although the predominant route of *blaTEM* transfer in *P. aeruginosa* remains conjugative plasmids and transposons (Stokes and Hall 1989; Poirel et al. 2010). The negligible association of *blaOXA‐48* with *IntI1* (*φ* = −0.04) confirms that its dissemination is predominantly integron‐independent, likely mediated through conjugative plasmids or insertion sequences, consistent with established mechanisms of carbapenemase gene transfer in *P. aeruginosa* (Stokes and Hall 1989; Poirel et al. 2010). Since *IntI2* and *IntI3* were entirely absent from this collection, no ARG–MGE associations involving these integron classes were observed or interpretable.

### 4.5 Pervasive Biofilm‐Forming Capacity Amplifies the Nosocomial Persistence Threat of MDR Environmental *P. aeruginosa*

The near‐universal biofilm‐forming capacity documented in this study is consistent with the established biology of *P. aeruginosa* as one of the most proficient biofilm‐producing pathogens among hospital‐associated microorganisms (Moradali et al. 2017; Sharma et al. 2019). Published literature consistently reports biofilm production in 75%–99% of *P. aeruginosa* isolates, with strong biofilm formation characterizing 8%–50% across clinical and environmental collections (Gajdács et al. 2021), placing the present findings well within the expected range. A study on MDR *P. aeruginosa* from hospital wastewater in Dhaka, Bangladesh similarly documented biofilm formation in 71 of 76 (93.4%) MDR isolates (Khan et al. 2024), reinforcing the conclusion that biofilm‐forming *P. aeruginosa* constitutes a persistent environmental hazard within Bangladeshi healthcare settings. The mechanisms underpinning this capacity, including quorum sensing‐regulated exopolysaccharide production, extracellular DNA secretion, and alginate overexpression, collectively render biofilm‐embedded cells tolerant to antibiotic concentrations up to 1000‐fold higher than planktonic counterparts and refractory to host immune clearance (Sharma et al. 2019; Ciofu et al. 2022).

The public health significance of these findings is amplified when biofilm formation is considered alongside the MDR phenotypes documented in Section 3.2. While several prior studies report no statistically significant association between biofilm‐forming capacity and antibiotic resistance in *P. aeruginosa* (Gajdács et al. 2021; Javanmardi et al. 2019), a growing body of evidence demonstrates that MDR and extensively drug‐resistant (XDR) lineages exhibit significantly elevated biofilm formation relative to susceptible strains, with biofilm identified as a key determinant of nosocomial persistence and endemic spread (Kaiser et al. 2017; Ghasemian et al. 2023). The biofilm matrix functions as a physical barrier reducing antibiotic penetration while simultaneously creating a microenvironment conducive to horizontal gene transfer, facilitating the exchange of resistance determinants between co‐resident strains and thereby amplifying the ARG and MGE dissemination dynamics documented in Sections 3.3 and 3.4 (Zafer et al. 2024; Salem et al. 2024). *P. aeruginosa* biofilms have been specifically implicated in ventilator‐associated pneumonia, surgical site infections, catheter‐associated urinary tract infections, and burn wound infections, all infection types directly associated with the high‐risk clinical units sampled in this study (Moradali et al. 2017; de Abreu et al. 2014).

The persistence of *P. aeruginosa* in hospital environments through biofilm formation on surfaces, water systems, and medical devices is well documented, with longitudinal studies demonstrating repeated isolation of the same clonal strains from hospital taps, sinks, and ward surfaces over periods of months to years (Trautmann et al. 2005; Hota et al. 2009). The high biofilm‐forming capacity of isolates recovered from the Operation Theatre, Pathology Unit, Burn Unit, and Outdoor Unit, combined with their MDR profiles and integron‐associated resistance gene carriage, collectively identify these sites as high‐priority targets for enhanced environmental decontamination and infection control intervention.

### 4.6 Association Between *lecB* ‐Based Strain Family Classification and Biofilm Formation

Comparison of *lecB*‐based strain family classification with quantitative biofilm production showed that strong biofilm‐forming isolates were proportionally more common within the PAO1‐like strain family (36%) than within the PA14‐like strain family (25%). However, this observation should be interpreted as descriptive because biofilm biomass did not differ significantly between the two groups (Mann–Whitney *U* test, *p* = 0.894), and the PA14‐like strain family was represented by only eight isolates. Consequently, the present data do not support a statistically significant association between *lecB*‐based strain family classification and biofilm‐forming capacity.

The biological relevance of the *lecB* gene nevertheless provides an important context for these observations. LecB is a fucose—and mannose‐binding lectin that contributes to bacterial adhesion, biofilm development, and virulence in *P. aeruginosa* , and experimental disruption of LecB has been shown to impair biofilm formation under both static and flow conditions (Moradali et al. 2017; Sharma et al. 2019). Moreover, Sommer et al. demonstrated that variation in the *lecB* sequence reliably distinguishes PAO1‐like and PA14‐like strain families and that the LecB variant from PA14 exhibits generally higher affinities for several carbohydrate ligands than the PAO1 variant, despite both variants displaying similar glycan‐binding specificities. They further concluded that the *lecB* sequence serves as a genetic marker for strain family classification while noting that the evolutionary and functional significance of these sequence differences requires further investigation.

Although differences in LecB ligand‐binding properties could potentially influence bacterial adhesion or biofilm development, the present findings do not demonstrate that *lecB* sequence variation alone determines biofilm‐forming capacity. Biofilm formation in *P. aeruginosa* is a complex phenotype controlled by numerous interacting factors, including exopolysaccharide production, quorum sensing, cyclic‐di‐GMP signalling, type IV pili, flagella, and multiple environmental conditions. Consequently, the heterogeneous biofilm phenotypes observed within both PAO1‐like and PA14‐like strain families are likely to reflect the combined effects of these regulatory networks rather than variation in *lecB* alone.

The predominance of moderate biofilm formers within the PA14‐like strain family (62%) should likewise be interpreted cautiously. This subgroup comprised only eight isolates, limiting statistical power, and therefore should not be regarded as representative of PA14‐like strains in general. Likewise, although the reference strain PA14 is recognized as highly virulent in experimental infection models, its pathogenicity reflects numerous virulence determinants beyond LecB, including accessory genomic elements and additional virulence‐associated genes. Accordingly, biofilm phenotype cannot be inferred solely from *lecB*‐based strain family classification.

Overall, the present study supports the utility of *lecB* sequencing as a practical molecular approach for assigning environmental *P. aeruginosa* isolates to the major PAO1‐like and PA14‐like strain families. However, the absence of a significant association between strain family classification and biofilm biomass indicates that *lecB* sequence analysis alone is insufficient for predicting biofilm‐forming capacity. Future studies involving larger and more balanced collections, together with multilocus or whole‐genome sequencing, will be necessary to determine whether broader genomic background contributes to variation in biofilm formation and environmental persistence among hospital‐associated *P. aeruginosa* isolates.

## 5 Conclusion

The presence of multidrug‐resistant, integron‐harbouring, biofilm‐forming *P. aeruginosa* across routine hospital surfaces and environments signals that resistance is no longer confined to the clinic—it is being sustained, amplified, and redistributed through the environment itself. If left unaddressed, such reservoirs will continue to silently fuel treatment failures, compromise surgical and critical care outcomes, and accelerate the erosion of last‐resort antibiotic options. Bridging this gap demands that hospital infection control evolve beyond patient‐centered surveillance to actively monitor and intervene in the environmental niches where resistance ecology is shaped. The study is also constrained by a limited ARG screening panel; the absence of carbapenemase genes beyond *blaOXA‐48*, efflux pump genes, and QRDR mutations from the PCR screen means that the full molecular basis of the observed resistance phenotypes, particularly ceftazidime and meropenem resistance, remains incompletely characterized.

## Author Contributions

**S. M. Kador:** conceptualization, methodology, formal analysis, visualization, writing – original draft. **Khandaker Adil:** methodology, formal analysis, data curation. **Md. Tanvir Islam:** conceptualization, investigation, funding acquisition, writing – review and editing. **Md. Mustak Ahmed:** methodology, validation, formal analysis. **Tanay Chakrovarty:** methodology, writing – review and editing, conceptualization, investigation, formal analysis. **Md. Mohsin Kobir:** validation, data curation. **Ovinu Kibria Islam:** conceptualization, investigation, funding acquisition, writing – review and editing, project administration, supervision, resources, validation. **Mousufa Akter:** methodology, data curation. **Tanzima Sharmim Taowhid:** methodology, data curation. **Md. Hasanuzzaman:** visualization, writing – review and editing, data curation, formal analysis.

## Funding

This work was supported by University Grants Commission of Bangladesh. Jashore University of Science and Technology Research Cell, 23‐FOBST‐03, 24‐FOBST‐05.

## Ethics Statement

This study was conducted exclusively on environmental samples (surface swabs and water samples) collected from hospital premises and did not involve human subjects, patient samples, or clinical specimens. Environmental sampling was carried out with the knowledge and cooperation of the respective hospital authorities. Accordingly, formal ethics committee approval was not required under applicable national guidelines. No personally identifiable information was collected at any stage of the study.

## Conflicts of Interest

The authors declare no conflicts of interest.

## Supporting information


**Supporting Information: 1** Biochemical Tests. Results of 32 standard biochemical tests performed on all 36 *Pseudomonas aeruginosa* isolates. Each row represents a test (e.g., oxidase, urease, TSI, pigment production) and each column an isolate, with positive (+) or negative (−) outcomes used for presumptive species identification.

**Supporting Information: 2** Media and Reagents. A catalogue of all culture media and chemical reagents used during biochemical characterization of the 36 isolates. Entries include the product name, BD brand/line, catalogue number, format, purpose, and storage conditions, organized by category (isolation media, differential media, and reagents).

**Supporting Information: 3** Antibiogram. Disk diffusion susceptibility results for all 36 isolates tested against eight antibiotics (aztreonam, meropenem, cefepime, gentamicin, tetracycline, tobramycin, ceftazidime, and levofloxacin). Each cell reports the zone diameter (mm) and the corresponding interpretation (R/I/S) based on CLSI breakpoints.

**Supporting Information: 4** PCR, Antibiotic, and MGE Results. Binary PCR screening results (+/−) for five resistance and mobile genetic element (MGE)‐associated genes—*bla*SHV, *bla*OXA‐48, *bla*TEM, *floR*, and *aac*(*6′*)*‐Ib*—across all 36 isolates, used to assess resistance gene carriage and co‐occurrence patterns.

**Figure S1:** Gram Staining Test.
**Figure S2:** KIA Test.
**Figure S3:** Oxidase Test.
**Figure S4:** PCR Identification of AMR Genes.
**Figure S5:** PCR Identification of Mobile Genetic Element.
**Figure S6:** Antibiogram Test.
**Figure S7:** Biofilm Test.

**Table S1:** Distribution of environmental samples collected across three hospitals and two sampling rounds.

**Table S2:** Primers used for PCR detection of antimicrobial resistance genes and mobile genetic elements and *lecB* gene detection.

## Data Availability

The nucleotide sequences generated in this study, including *lecB* Sanger sequencing data and whole genome sequencing reads for the seven representative isolates, have been deposited in the NCBI database under BioProject accession number PRJNA1464230. All other data supporting the findings of this study are available within the manuscript and its Supporting Information.
