# Supplementary material for: Occurrence and Distribution of Environmental Pseudomonas aeruginosa From Hospitals in Bangladesh Reveals Diverse Strain Families, Multidrug Resistance, and Biofilm Formation
Source: Environ Microbiol Rep. 2026 Jul 24;18(4):e70391. doi: 10.1111/1758-2229.70391 (PMC13400180; doi:10.1111/1758-2229.70391)
Supplement: Supplementary file 5 — Figure S1: Gram Staining Test. Figure S2: KIA Test. Figure S3: Oxidase Test. Figure S4: PCR Identification of AMR Genes. Figure S5: PCR Identification of Mobile Genetic Element. Figure S6: Antibiogram Test. Figure S7: Biofilm Test. [file EMI4-18-e70391-s003.docx]

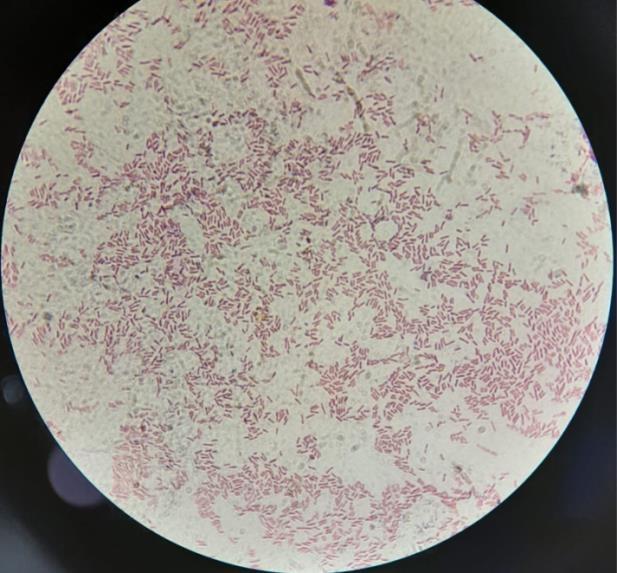


Gram Negative Rods

Fig. S1 – Gram Staining Test


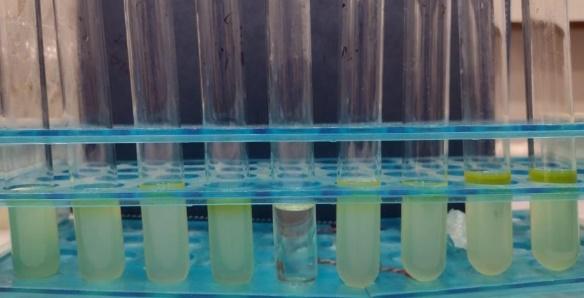


Glucose positive

lactose positive

Control

Fig. S2 – KIA Test

Positive


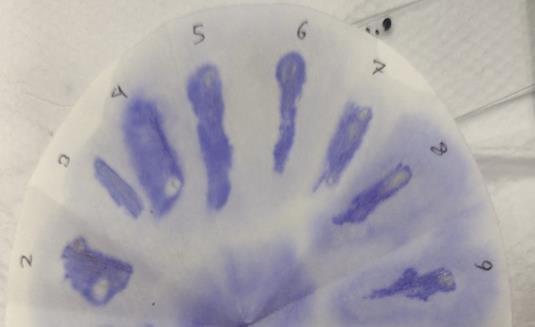


Fig

Fig. S3 – Oxidase Test


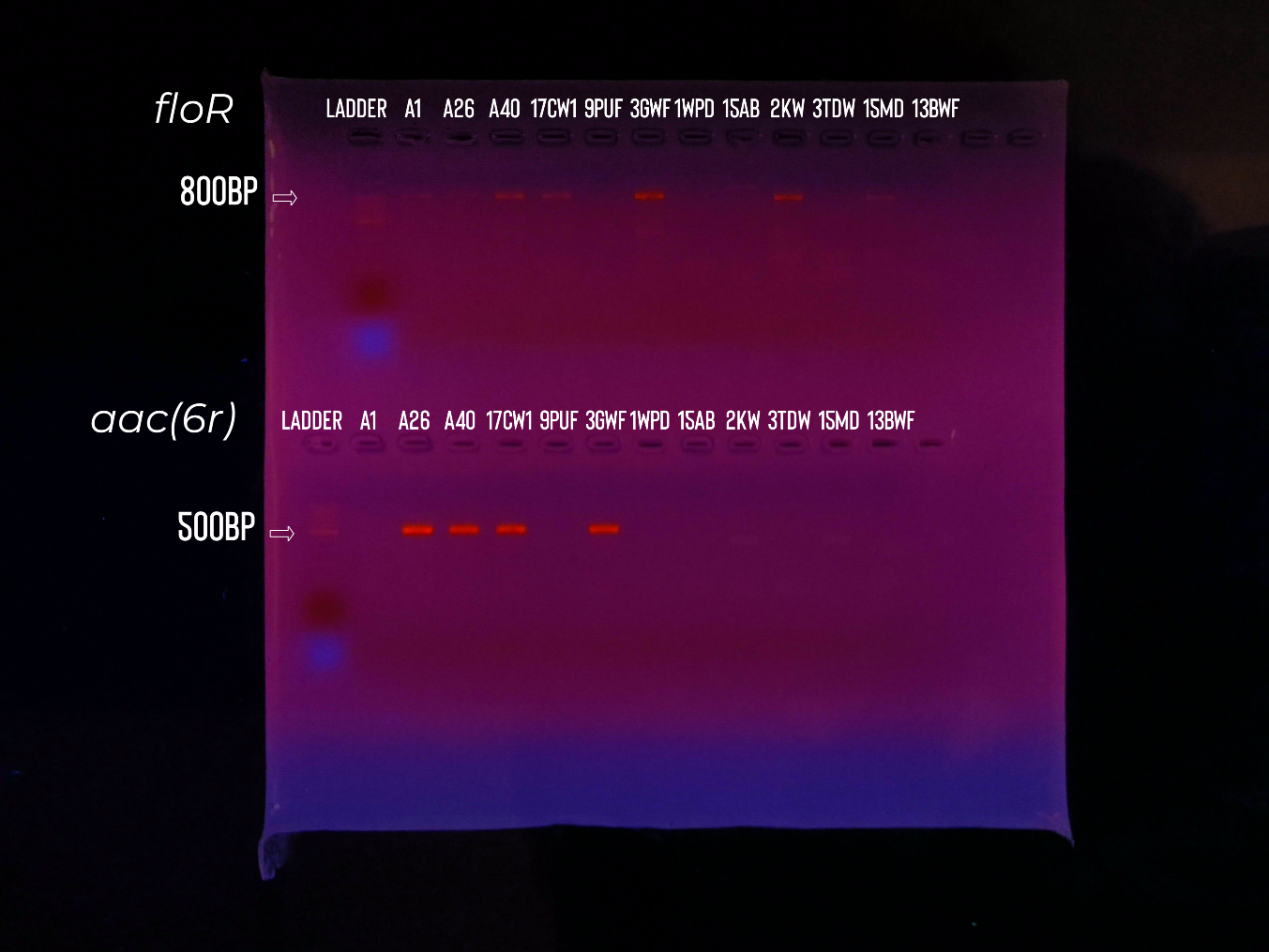


100bp

100bp

Fig. S4 – PCR Identification of AMR Genes


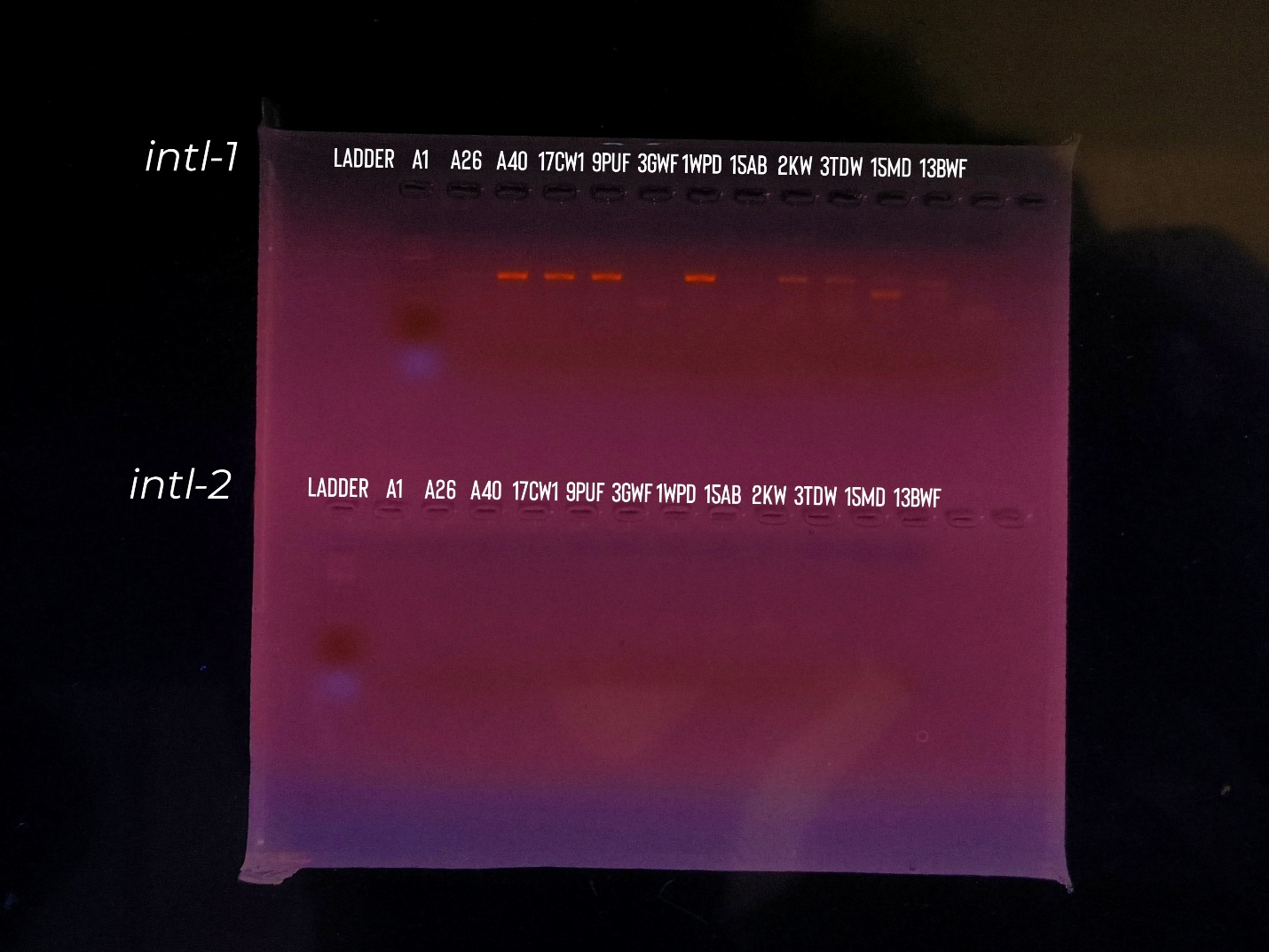


Fig. S5 - PCR Identification of Mobile Genetic Element


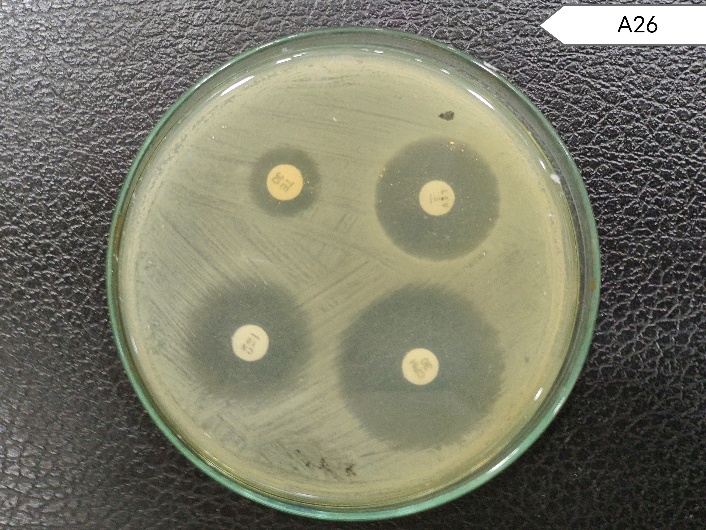

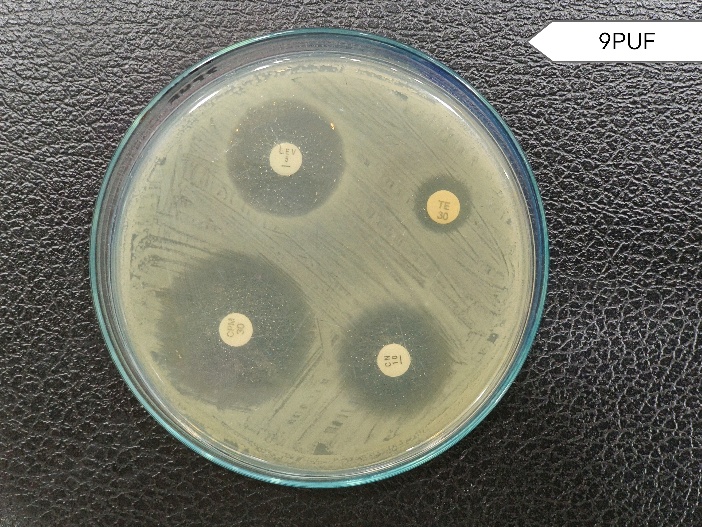


Tetracycline

Levofloxacin

Levofloxacin

Tetracycline

Gentamicin

Cefepime

Gentamicin

Cefepime

Fig. S6 – Antibiogram Test


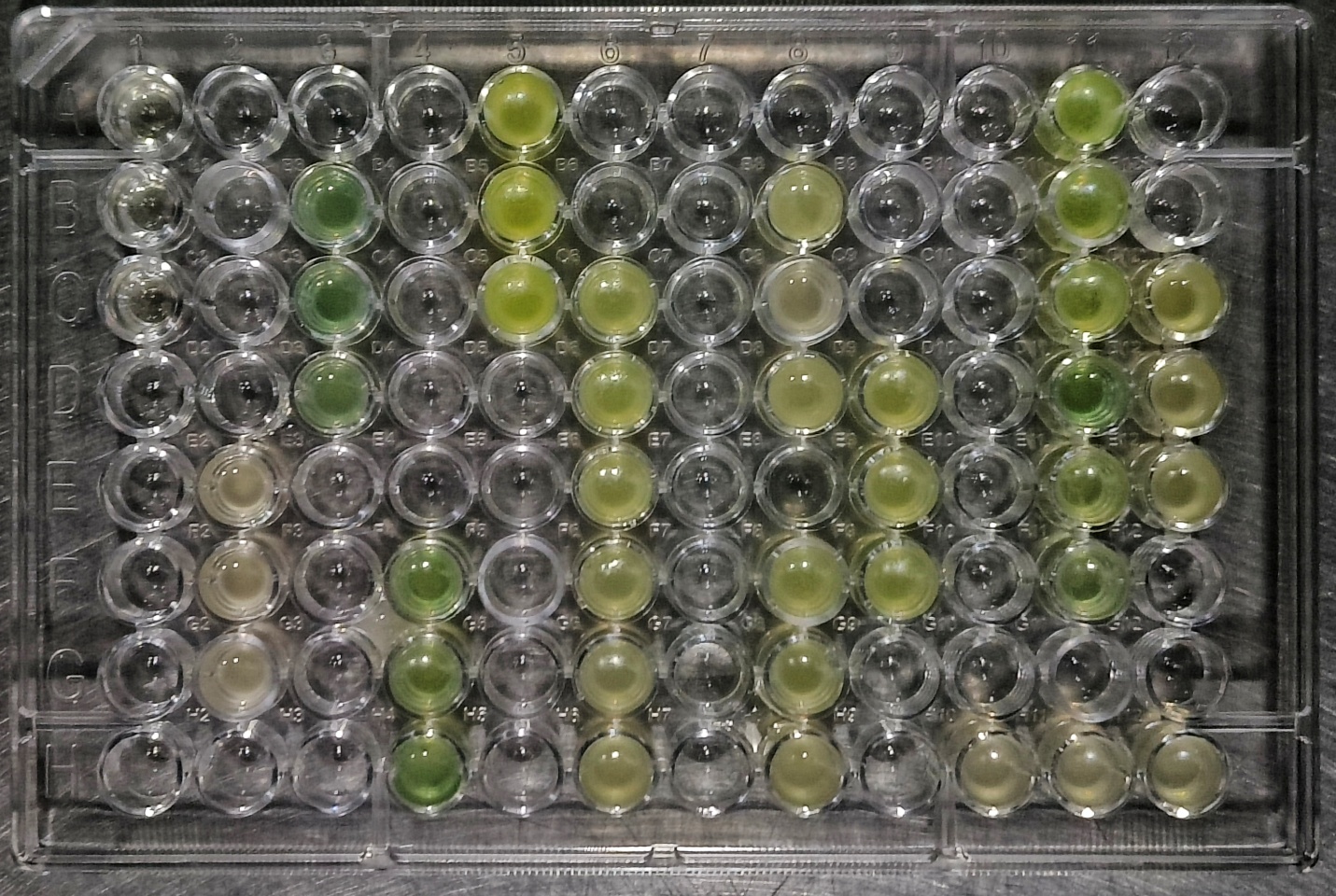


Strong Biofilm Former

Control

Weak Biofilm Former

Fig. S7 – Biofilm Test
