# Supplementary material for: Occurrence and Distribution of Environmental Pseudomonas aeruginosa From Hospitals in Bangladesh Reveals Diverse Strain Families, Multidrug Resistance, and Biofilm Formation
Source: Environ Microbiol Rep. 2026 Jul 24;18(4):e70391. doi: 10.1111/1758-2229.70391 (PMC13400180; doi:10.1111/1758-2229.70391)
Supplement: Supplementary file 6 — Table S1: Distribution of environmental samples collected across three hospitals and two sampling rounds. [file EMI4-18-e70391-s007.docx]

# Supplementary Table S1

Distribution of environmental samples collected across three hospitals and two sampling rounds

| **Sl No.** | **Sampling Site** | **Hospital 1 (250-Seated)** | | | **Hospital 2**  **(Queens)** | | | **Hospital 3 (Ibn Sina)** | | | **Total Samples** | **No. of P. aeruginosa isolates** | **Isolate ID (WGS isolates in bold italic)** |
| --- | --- | --- | --- | --- | --- | --- | --- | --- | --- | --- | --- | --- | --- |
|  |  | **R1*** | **R2**** | **Total** | **R1*** | **R2**** | **Total** | **R1*** | **R2**** | **Total** |  |  |  |
| 1 | Surgical unit | 5 | 7 | 12 | 5 | 4 | 9 |  |  |  | 21 | 1 | ***PD*** |
| 2 | I.C.U unit | 2 | 2 | 4 |  | 1 | 1 |  |  |  | 5 |  |  |
| 3 | Pneumonia ward |  | 1 | 1 | 6 | 2 | 8 |  |  |  | 9 | 2 | 7PWW, PWW |
| 4 | Pathology unit | 6 | 4 | 10 |  | 1 | 1 | 3 | 1 | 4 | 15 | 1 | ***9PUF*** |
| 5 | Kitchen water | 3 | 1 | 4 | 1 |  | 1 | 1 |  | 1 | 6 |  |  |
| 6 | Corridor | 2 |  | 2 |  | 5 | 5 |  |  |  | 7 |  |  |
| 7 | Outdoor unit | 2 |  | 2 |  | 3 | 3 |  |  |  | 5 | 2 | ***2OB***, OB |
| 8 | Autoclave room | 2 |  | 2 |  |  |  |  |  |  | 2 |  |  |
| 9 | Male ward | 8 | 2 | 10 | 1 | 1 | 2 | 6 | 4 | 10 | 22 | 1 | ***15MD*** |
| 10 | Ambulance |  |  |  |  |  |  | 1 | 1 | 2 | 2 | 2 | 15AB, A1 |
| 11 | Toilet |  | 3 | 3 | 2 | 4 | 6 | 1 | 1 | 2 | 11 | 2 | MTW, ***FT*** |
| 12 | Toilet Water | 2 | 5 | 7 |  |  |  |  |  |  | 7 | 2 | 3TDW, TW |
| 13 | Female ward | 1 |  | 1 |  | 4 | 4 |  |  |  | 5 | 2 | ***FWD***, FW |
| 14 | Gynae ward | 2 | 1 | 3 | 2 |  | 2 |  | 1 | 1 | 6 | 1 | 3GWF |
| 15 | Wound patients ward |  | 1 | 1 | 2 | 2 | 4 |  | 1 | 1 | 6 | 3 | 1WPD, WPF, ***WPF1*** |
| 16 | Drain |  | 2 | 2 | 1 | 1 | 2 |  | 1 | 1 | 5 | 2 | DW, HD |
| 17 | Kidney patients ward | 2 |  | 2 |  | 1 | 1 |  |  |  | 3 | 1 | 2KW |
| 18 | Burn unit | 2 | 1 | 3 | 1 |  | 1 |  |  |  | 4 | 4 | 13BWF, BW1, BW2, ***BW3*** |
| 19 | Diabetes ward |  | 3 | 3 |  |  |  |  |  |  | 3 |  |  |
| 20 | Asthma ward |  | 1 | 1 |  |  |  | 1 |  | 1 | 2 | 1 | A26 |
| 21 | Plaster room |  |  |  | 2 | 2 | 4 |  |  |  | 4 |  |  |
| 22 | Dustbin |  | 2 | 2 |  | 2 | 2 |  | 1 | 1 | 5 |  |  |
| 23 | Entrance | 6 | 8 | 14 | 2 |  | 2 |  | 3 | 3 | 19 |  |  |
| 24 | X-Ray |  | 4 | 4 |  |  |  |  |  |  | 4 |  |  |
| 25 | Operation Theatre | 2 |  | 2 | 3 | 3 | 6 | 1 | 6 | 7 | 15 | 6 | A40, 2OTI, 2SW, 3OTF, OTW, SW |
| 26 | Lift |  | 1 | 1 |  | 2 | 2 | 3 | 1 | 4 | 7 |  |  |
| 27 | Stretcher | 2 |  | 2 |  |  |  |  | 1 | 1 | 3 |  |  |
| 28 | Pediatric Ward | 2 | 2 | 4 | 3 |  | 3 | 3 | 7 | 10 | 17 | 3 | 17CW1, CWF2 |
| 29 | CCU | 2 |  | 2 | 3 | 6 | 9 |  |  |  | 11 |  | CCUW |
|  | **Total** |  |  | **104** |  |  | **78** |  |  | **49** | **231** | **36** |  |

** Round 1: April–August 2025*

*** Round 2: August–November 2025*

*250-Seated General Hospital Jashore (Hospital 1), Queens Hospital Jashore (Hospital 2) and Ibn Sina Hospital Jashore (Hospital 3)*
