# Supplementary material for: Occurrence and Distribution of Environmental Pseudomonas aeruginosa From Hospitals in Bangladesh Reveals Diverse Strain Families, Multidrug Resistance, and Biofilm Formation
Source: Environ Microbiol Rep. 2026 Jul 24;18(4):e70391. doi: 10.1111/1758-2229.70391 (PMC13400180; doi:10.1111/1758-2229.70391)
Supplement: Supplementary file 7 — Table S2: Primers used for PCR detection of antimicrobial resistance genes and mobile genetic elements and lecB gene detection. [file EMI4-18-e70391-s006.docx]

**Table S2:** Primers used for PCR detection of antimicrobial resistance genes and mobile genetic elements and *lecB* gene detection

| **Gene** | **Primer Sequence (5'→3')** | **Amplicon Size (bp)** | **Annealing Temp (°C)** | **Reference** |
| --- | --- | --- | --- | --- |
| *blaSHV* | F: GGTTATGCGTTATATTCGCC | 868 | 55 | [8] |
|  | R: TTAGCGTTGCCAGTGCTC |  |  |  |
| *blaOXA-48* | F: GCGTGGTTAAGGATGAACAC | 438 | 53 | [9] |
|  | R: CATCAAGTTCAACCCAACCG |  |  |  |
| *blaTEM* | F: ATGAGTATTCAACATTTCCG | 858 | 55 | [8] |
|  | R: CCAATGCTTAATCAGTGAGG |  |  |  |
| *floR* | F: ACCTGGAGCGATAACCTTGT | 530 | 57 | [14] |
|  | R: GCACCAATCAGGTTGATGTC |  |  |  |
| *aac(6r)* | F: AGTGTTGGCGATTATCTTGG | 286 | 54 | [10] |
|  | R: CTTCACGAATTTGCCTGATTG |  |  |  |
| *IntI1* | F: GGGTCAAGGATCTGGATTTCG | 483 | 55 | [12] |
|  | R: ACATGCGTGTAAATCATCGTC |  |  |  |
| *IntI2* | F: CACGGATATGCGACAAAAAGGT | 788 | 55 | [12] |
|  | R: GTAGCAAACGAGTGACGAAATG |  |  |  |
| *IntI3* | F: GCCTCCGGCAGCGACTTTCAG | 979 | 58 | [13] |
|  | R: ACGGATCTGCCAAACCTGACT |  |  |  |
| *lecB* | F: ATGAACAACCTTTTCAGCCTG | 287 | 55 | [28] |
|  | R: TCAGGCCTTGCCGAAGTC |  |  |  |

**References**

[8] Shahcheraghi F, Nikbin VS, Feizabadi MM. Detection of ESBLs types *blaCTX-M*, *blaSHV* and *blaTEM* resistance genes among clinical isolates of *Pseudomonas aeruginosa*. *New Microbes New Infect*. 2022;47:100980.

[9] Abdelraheem WM, El-Mahdy RI, Ibrahim AM, Hammad SS. Prevalence of *blaOXA-48* and other carbapenemase encoding genes among carbapenem-resistant *Pseudomonas aeruginosa* clinical isolates in Egypt. *BMC Infect Dis*. 2024;24(1):1212.

[10] Teixeira B, Rodulfo H, Carreño N, Guzmán M, Salazar E, De Donato M. Aminoglycoside resistance genes in *Pseudomonas aeruginosa* isolates from Cumana, Venezuela. *Rev Inst Med Trop Sao Paulo*. 2016;58:13.

[12] Khademi F, Vaez H, Sahebkar A. Prevalence of class I, II and III integrons in multidrug-resistant and carbapenem-resistant *Pseudomonas aeruginosa* clinical isolates. *New Microbes New Infect*. 2021;44:100944.

[13] Zahedi Bialvaei A, Samadi Kafil H, Ebrahimzadeh Leylabadlo H, Asgharzadeh M, Aghazadeh M. Prevalence of integrons 1, 2, 3 associated with antibiotic resistance in *Pseudomonas aeruginosa* isolates from northwest of Iran. *Biomed J*. 2018;41(3):162–167.

[14] Chen J, Jin M, Qiu ZG, et al. A survey of drug resistance *bla* genes originating from synthetic plasmid vectors in Chinese rivers. *Environ Sci Technol*. 2012;46(24):13448–13454.

[28] Tielker, D., et al., Pseudomonas aeruginosa lectin LecB is located in the outer membrane and is involved in biofilm formation. 2005. 151(5): p. 1313-1323.
